# ProteomeLM: A proteome-scale language model enables accurate and rapid prediction of protein–protein interactions and gene essentiality across taxa

**DOI:** 10.1073/pnas.2524201123

**Published:** 2026-05-20

**Authors:** Cyril Malbranke, Gionata Paolo Zalaffi, Anne-Florence Bitbol

**Affiliations:** ^a^ Institute of Bioengineering, School of Life Sciences, École Polytechnique Fédérale de Lausanne (EPFL), Lausanne CH-1015, Switzerland; ^b^ Swiss Institute of Bioinformatics, Lausanne CH-1015, Switzerland

**Keywords:** protein sequences, protein–protein interactions, gene essentiality, coevolution, biological language models

## Abstract

Language models trained on biological sequences are advancing inference tasks from the scale of single proteins to that of genomic neighborhoods. Here, we introduce ProteomeLM, a transformer-based language model that uniquely operates on entire proteomes from species spanning the tree of life. ProteomeLM is trained to reconstruct masked protein embeddings using the whole proteomic context, yielding contextualized protein representations that reflect proteome-scale functional constraints. Notably, ProteomeLM’s attention coefficients encode protein–protein interactions (PPI), despite being trained without interaction labels. Furthermore, it enables interactome-wide PPI screening that is substantially more accurate, and orders of magnitude faster, than amino acid coevolution-based methods. We further develop ProteomeLM-PPI, a supervised model that combines ProteomeLM embeddings and attention coefficients to achieve state-of-the-art PPI prediction across benchmarks and species. Finally, we introduce ProteomeLM-Ess, a supervised gene essentiality predictor that generalizes across diverse taxa. Our results demonstrate the potential of proteome-scale language models for addressing function and interactions at the organism level.

## Significance

Predicting protein interactions and functions is a key challenge in biology. Although deep learning-based language models are advancing the analysis of individual protein sequences and of genomic neighborhoods, they struggle to capture properties involving all the proteins expressed in a cell, such as protein–protein interactions (PPI) and gene essentiality. We present ProteomeLM, a language model that reasons on entire proteomes across diverse species. ProteomeLM captures PPI without supervision, and enables more accurate and faster screening of entire interactomes than current sequence-based approaches. ProteomeLM also delivers state-of-the-art supervised PPI prediction, and improves supervised prediction of gene essentiality compared to protein language models. These results demonstrate the potential of proteome-scale language models to reveal system-level organization and functional relationships.

Recently, deep learning approaches have brought important progress to inference from biological sequence data. Protein language models are deep learning models based on natural language processing methods. Trained on large ensembles of protein sequences, they learn sequence representations that encode structural and functional signals (1–12), and have advanced the prediction of protein structure (9, 10), subcellular localization (13), and mutational effects (14, 15). Similarly, genome language models (16–26) have given insight into noncoding DNA, gene expression, and taxonomic classification (17–19), capturing operons, enzymatic function (21) and within-operon protein–protein interactions (PPI) (22), and predicting mutation effects (20). However, so far, these models span at most hundreds of kilobases or a few megabases (18, 19, 23–26). As they do not capture dependencies across entire genomes, especially in eukaryotes, these models cannot predict system-level properties such as PPI involving distant genomic regions.

PPI are fundamental to most biological processes, including signal transduction, cellular metabolism, and immune responses. Knowing these interactions is critical for deciphering cellular processes and for developing therapeutic interventions. However, large-scale PPI determination—up to the full PPI networks of nonmodel species—remains a significant challenge (27). Indeed, precise experimental methods are both labor-intensive and costly, particularly when scaled to entire proteomes, and high-throughput ones have limited accuracy (28). While curated PPI databases have grown (29–31), they remain incomplete and biased toward well-studied species and interaction types. Computational PPI predictions have been developed to overcome this gap. Structure-based ones, including docking (32–35) and multimeric folding algorithms like AlphaFold-Multimer (36), have achieved remarkable accuracy for specific interactions (37), but remain computationally intensive. Sequence-based methods, which rely on evolutionary signals, offer better scalability. Amino acids that are in contact at the interface of protein complexes need to maintain physico-chemical complementarity through evolution, yielding correlations in amino acid usage at contacting sites, known as coevolution (38–41). This signal can be detected by Potts models, also known as direct coupling analysis (DCA), which are trained on multiple sequence alignments of homologous proteins for each candidate PPI (42–48). At a larger scale, during evolution, genes coding for proteins that interact tend to undergo similar evolutionary pressures and to be either present or absent together in each genome, thus having correlated co-occurrence patterns across genomes. This coevolution between genes is exploited by phylogenetic profiling (49–57) and co-occurrence methods (58). While such coevolution methods are often effective in bacteria and other well-represented clades, they struggle in eukaryotes or poorly sampled taxa, and require careful curation of orthologs, and for DCA, paired multiple sequence alignments (45, 46, 59, 60).

Protein language models trained with the masked language modeling (MLM) objective of predicting masked amino acids in sequences using the surrounding context (3, 9–12) learn coevolution between amino acids. This allows them, in particular those based on multiple sequence alignments (9), to capture protein structure, an ability exploited in AlphaFold2 via its EvoFormer module (61). Given the success of protein language models at capturing coevolution between amino acids, it is tantalizing to develop such models at the proteome scale, i.e. taking as input the ensemble of proteins encoded by a genome. Indeed, we posit that such models should capture coevolution between proteins, thereby generalizing over phylogenetic profiling methods (49–57). This should make them highly suited to provide predictions of complete PPI networks, and of their evolution. Importantly, once trained, the computational cost of prediction on new species should be small. Moreover, such foundation models can also be used for other downstream applications where proteome context information is important, such as predicting gene essentiality (62).

In this paper, we introduce ProteomeLM, a transformer-based language model that uniquely reasons on entire proteomes from multiple species spanning the tree of life. ProteomeLM leverages embeddings from the protein language model ESM-Cambrian (12), and is thus informed of functional protein-level properties, which it integrates at the proteome scale. We show that ProteomeLM’s attention coefficients learn PPI in an unsupervised way. These PPI include broad functional relationships and protein complex membership, which yield distinct signals. Building on this ability of ProteomeLM, we propose a method to screen whole interactomes orders of magnitude faster than DCA pipelines, and we demonstrate that it substantially outperforms them. Next, we introduce ProteomeLM-PPI, a supervised PPI prediction network that combines ProteomeLM embeddings and attention coefficients, and we show that it achieves state-of-the-art supervised PPI prediction across species and benchmarks. Finally, as another example of downstream tasks allowed by ProteomeLM embeddings, we introduce ProteomeLM-Ess, a supervised predictor of gene essentiality that generalizes across diverse taxa. Our results show that representing proteins in their full proteome context allows ProteomeLM to capture system-level biological signals that remain inaccessible to models that reason on individual proteins or on local genomic contexts.

## Results

### A Proteome-Scale Language Model Leveraging Protein Language Model Representations.

We introduce ProteomeLM, a transformer-based proteome language model (LM). We trained ProteomeLM on a large corpus of proteomes, spanning the tree of life, from bacteria and archaea to eukaryotes and viruses, to learn protein representations in the context of complete proteomes. ProteomeLM takes as input a proteome, i.e., the set of proteins encoded by a given genome, and aims to capture the functional and evolutionary signals present between proteins at the proteome level.

**Fig. 1.**
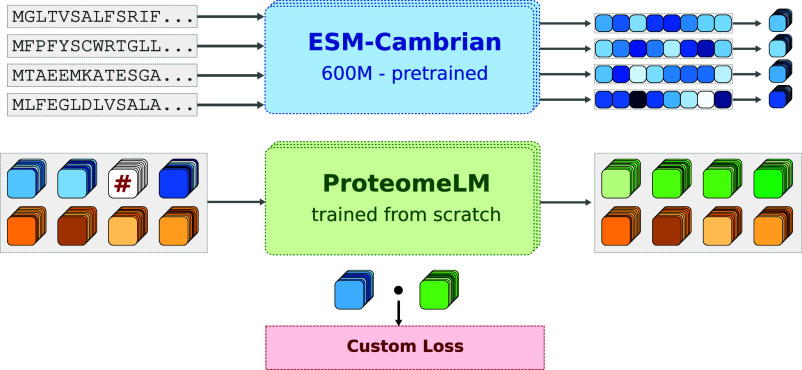
ProteomeLM training. Input amino acid sequences, corresponding to proteins encoded by a genome, are embedded through the pretrained ESM-C model, yielding a fixed-dimensional embedding for each protein. The embeddings serve as input to ProteomeLM, trained from scratch to predict the masked embeddings of proteins in the context of their proteome (*Materials and Methods*). Masked embeddings are indicated by “#” in the schematic. Proteins are annotated by a functional encoding (shown in orange below embeddings) representing their orthologous group. ProteomeLM’s training uses a custom polar loss (*Materials and Methods*): for each masked protein, it essentially aims to minimize the difference between the ESM-C embedding and the ProteomeLM embedding, but in a protein family-specific manner.

Specifically, we start from each protein’s amino acid sequence, and represent it by an embedding generated by the protein language model ESM-Cambrian (ESM-C) (12), see Fig. 1 and *Protein Representations*. This allows our model to leverage the rich functional sequence-derived properties (63, 64) learned for each protein by protein language models (3, 9, 10, 14) (see also refs. 21, 22, and 65). During training, a subset of these protein embeddings is masked, and the model is tasked with reconstructing them using the remaining unmasked protein embeddings from the same proteome, see Fig. 1. This masked language modeling prediction task allows ProteomeLM to learn the dependencies between proteins encoded by a given genome.

A challenge when constructing a model that can reason across diverse organisms is the fact that their genome organization is very different. For instance, while bacterial genes are organized in operons, functionally related proteins are often not encoded in proximity in eukaryotic genomes, and gene order is less conserved in eukaryotes than in prokaryotes. Therefore, ProteomeLM does not employ positional encoding along the genome, which sets it apart from existing genome language models (and protein language models). Instead, we propose a functional encoding, based on orthology, which captures shared evolutionary and functional relationships across genes in different genomes (*Functional Encoding*). Orthologous groups comprise genes in different species that descend from a common ancestral gene via speciation, and generally have retained the same function. Phylogenetic profiling methods have shown that the presence and absence data of orthologous groups, across genomes, contain information about functional relationships (49–51, 54, 66). For instance, genes coding for interacting proteins tend to have correlated presence–absence vectors across genomes, allowing to predict interactions. Hence, we posit that functional encoding will help ProteomeLM to learn coevolution between proteins in a proteome, and thus, their functional relationships. In practice, orthologous groups were collected from OrthoDB (67). Note that they were built using statistical homolog matching, and no human annotations.

ProteomeLM was trained on nearly 32,000 annotated proteomes, spanning all domains of life (*Dataset*). By learning to reconstruct masked proteins from their genomic context, ProteomeLM is encouraged to learn the relationships that define protein function and interactions. We trained versions of ProteomeLM with multiple sizes, ranging from 6M to 328M parameters. We observed stable learning dynamics in all cases (*Training Dynamics and Scaling Behavior*).

### ProteomeLM Attention Coefficients Capture PPI.

ProteomeLM was trained on full proteomes, and informed by protein-level properties, thanks to the use of embeddings from protein language models. Does it learn PPI by being trained to predict a protein’s embedding using the context of its proteome? To address this question, we examine the attention coefficients of the model (5, 9, 68). Indeed, in a transformer model, attention coefficients encode the importance of each part of the context (here, of each protein) when performing masked language modeling (here, when aiming to predict another masked protein). Thus, we hypothesize that these coefficients should capture functional dependencies between proteins, such as PPI. For each pair of protein in five species, we compare these attention coefficients to known PPI. The species considered are *Escherichia coli*, *Saccharomyces cerevisiae*, *Caenorhabditis elegans*, *Drosophila melanogaster*, *Mus musculus*, and *Homo sapiens*. Specifically, we use the D-SCRIPT dataset (27), which is derived from the STRING database (69), and focuses exclusively on experimentally validated physical interactions. Complete proteomes from the five species considered were processed through ProteomeLM, and we extracted attention coefficients for each positive and negative interaction pair across both datasets.

**Fig. 2.**
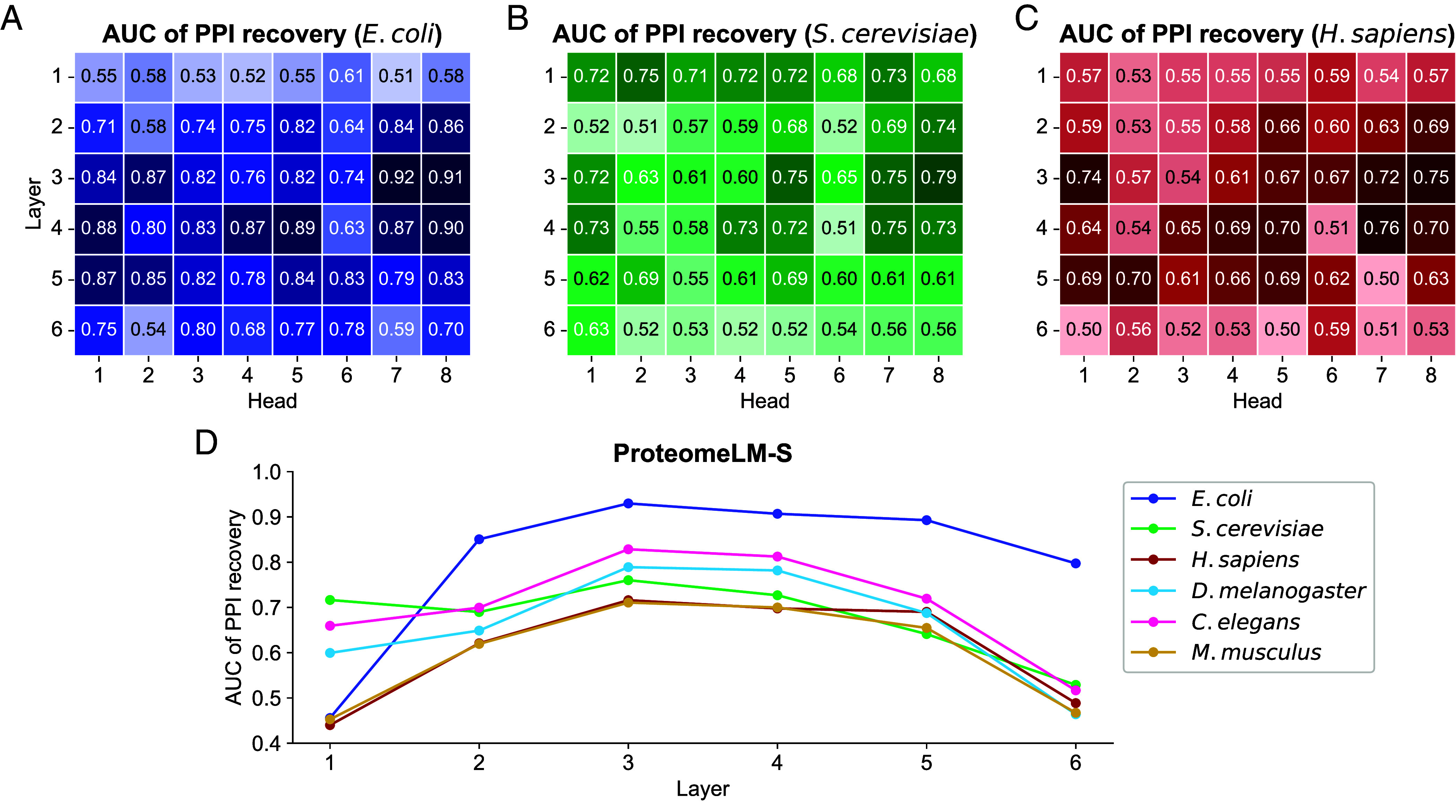
Unsupervised detection of PPI using ProteomeLM attention coefficients. We assess the ability of the attention coefficients of ProteomeLM-S (36M parameters) at predicting PPI from the D-SCRIPT dataset (27), using the area under the receiver operating characteristic curve (AUC) as a metric. (*A*–*C*) In three species, we report the AUC of each attention head in each layer of ProteomeLM, measuring its ability to distinguish interacting from noninteracting protein pairs (1: perfect classifier; 0.5: random). (*D*) We show the AUC obtained for the mean of attention coefficients over all heads in each layer of ProteomeLM-S, in each of the six species considered.

Fig. 2*A*–*C* shows that many attention heads of ProteomeLM are predictive of interaction labels. Moreover, we observe that several attention heads possess significant predictive power across all species considered (3 extra species are shown in *SI Appendix*, Fig. S6). In particular, head 7 of layer 3 achieves an AUC of 0.92 in *E. coli*, while also performing strongly in other species. The larger ProteomeLM-M also exhibits strong unsupervised PPI recovery, see *SI Appendix*, Fig. S7. Thus, ProteomeLM can identify PPI among vast numbers of possible protein pairs in a complete proteome (e.g., ∼4,000 proteins in *E. coli* and ∼20,000 in humans, leading to ∼8 $\times \;10^{6}$ and ∼2 $\times \;10^{8}$ possible pairs, respectively) in an unsupervised manner, without any fine-tuning. This is especially compelling given that ProteomeLM does not rely on gene order or local genomic context. The learning of PPI arises directly from the masked prediction training, which promotes the learning of dependencies between proteins in a proteome. Our result is reminiscent of the finding that protein language models’ attention coefficients carry information about residue–residue interactions involved in the three-dimensional structure of proteins, while being trained only on sequence data with the objective of filling in masked amino acids(3, 9, 10).

Fig. 2*D* further shows that PPI are most accurately captured by attention heads in the central layers of the model. A similar trend is observed across other model sizes (XS, M, and L), see *SI Appendix*, Fig. S8. In protein language models, central layers are known to capture more complex interactions than early layers (5). Thus, our finding supports the notion that higher-order interactions, rather than simple local features, are essential for understanding PPI, and that ProteomeLM can extract such complex biological signals.

We next ask what types of PPI are captured by ProteomeLM’s attention heads. As ProteomeLM learns statistical dependencies between proteins from their context across thousands of proteomes, and shares similarities with phylogenetic profiling (49), its attention coefficients are expected to capture broader functional associations (e.g., coregulation) beyond direct physical binding. To address this question, we build a benchmark distinguishing direct interactions [proteins that directly bind to each other, from PDB (70)], same-complex interactions (protein pairs found in the same complex but not directly binding, from PDB), and genetic associations [coexpression pairs from STRING (69)]. We find that ProteomeLM captures all three interaction types in its attention coefficients across *E. coli*, *S. cerevisiae* and *H. sapiens*. Genetic associations are recovered with higher accuracy (AUC≥0.92 in all three species) than direct or same-complex interactions (0.74≤AUC≤0.92, see *SI Appendix*, section 1). Moreover, a linear classifier based on its attention coefficients can distinguish direct interactions from genetic associations with high accuracy (AUC=0.89 in yeast, 0.90 in humans), although the signal separating direct from same-complex interactions is less strong (*SI Appendix*, section 1). Thus, ProteomeLM learns representations that disentangle physical binding from functional associations. As a specific test of ProteomeLM’s ability to distinguish direct interactions from same-complex interactions, we further consider two structurally resolved protein complexes: the *E. coli* ribosome and the *S. cerevisiae* TRiC/CCT chaperonin. In both cases, ProteomeLM excels at distinguishing protein pairs belonging to a given complex from the rest of the proteome (AUC≥0.99). Direct interactions within the complexes are more weakly captured, with statistically significant signal for the *E. coli* ribosome but not for the *S. cerevisiae* TRiC/CCT chaperonin (*SI Appendix*, section 2). The latter complex is particularly challenging, as all of its subunits descend from a common ancestor.

Overall, ProteomeLM is an excellent predictor of broad functional relationships and of protein complex membership. Furthermore, it outperforms its input ESM-C embeddings and functional encodings, which already encode evolutionary and functional information (*SI Appendix*, section 3).

### ProteomeLM Provides Fast and Accurate PPI Screening.

ProteomeLM successfully captures PPI, and was trained on organisms spanning the tree of life. Can it thus contribute to shedding light on complete interactomes in various species? Current large-scale interactome prediction workflows generally rely on a two-stage pipeline (48, 71, 72). First, relatively light sequence-based methods exploiting coevolution in multiple sequence alignments (MSAs), in particular Potts models, also known as direct coupling analysis (DCA) (42–44), are used to identify promising protein pairs that may interact. Second, heavier structure-based methods like AlphaFold-Multimer (36) or RoseTTAFold-PPI (72) are used to further analyze these candidate pairs, through computationally intensive structural modeling. While effective, the first step of this approach is limited by the computational cost of MSA generation, and by the sheer number of pairwise models required to scan large proteomes. Indeed, DCA is a family-specific model, so one model has to be trained per candidate protein pair. To this day, such large-scale PPI screens have thus only been performed on a few model organisms, namely *E. coli* (47) and *S. cerevisiae* (48), and very recently on *H. sapiens* (72), and on 19 human pathogens (71).

To assess ProteomeLM’s promise as a first filter for interactome prediction, we trained a lightly supervised and lightweight classifier on attention coefficients from ProteomeLM, and evaluated its performance on the full human interactome and across pathogen interactomes. We compared its computational requirements and its performance to those reported in a recent large-scale study that applied DCA respectively to over 190 million *H. sapiens* protein pairs (72). We also tested our method on 19 human bacterial pathogens considered in ref. 71, collectively comprising 102 million protein pairs. Specifically, our classifier is a logistic regression aiming to predict PPI from a combination of the 48 attention heads of ProteomeLM-S (Fig. 2*A*–*C*), and is trained over a small set of 100 interacting pairs and 1,000 random pairs (treated as noninteracting), which are later held out from evaluation. Positive pairs are sampled among the pairs that have a confidence score above 0.99 (extremely high) in the STRING database (69), either among human pairs or among pathogenic pairs in the corresponding datasets.

**Fig. 3.**
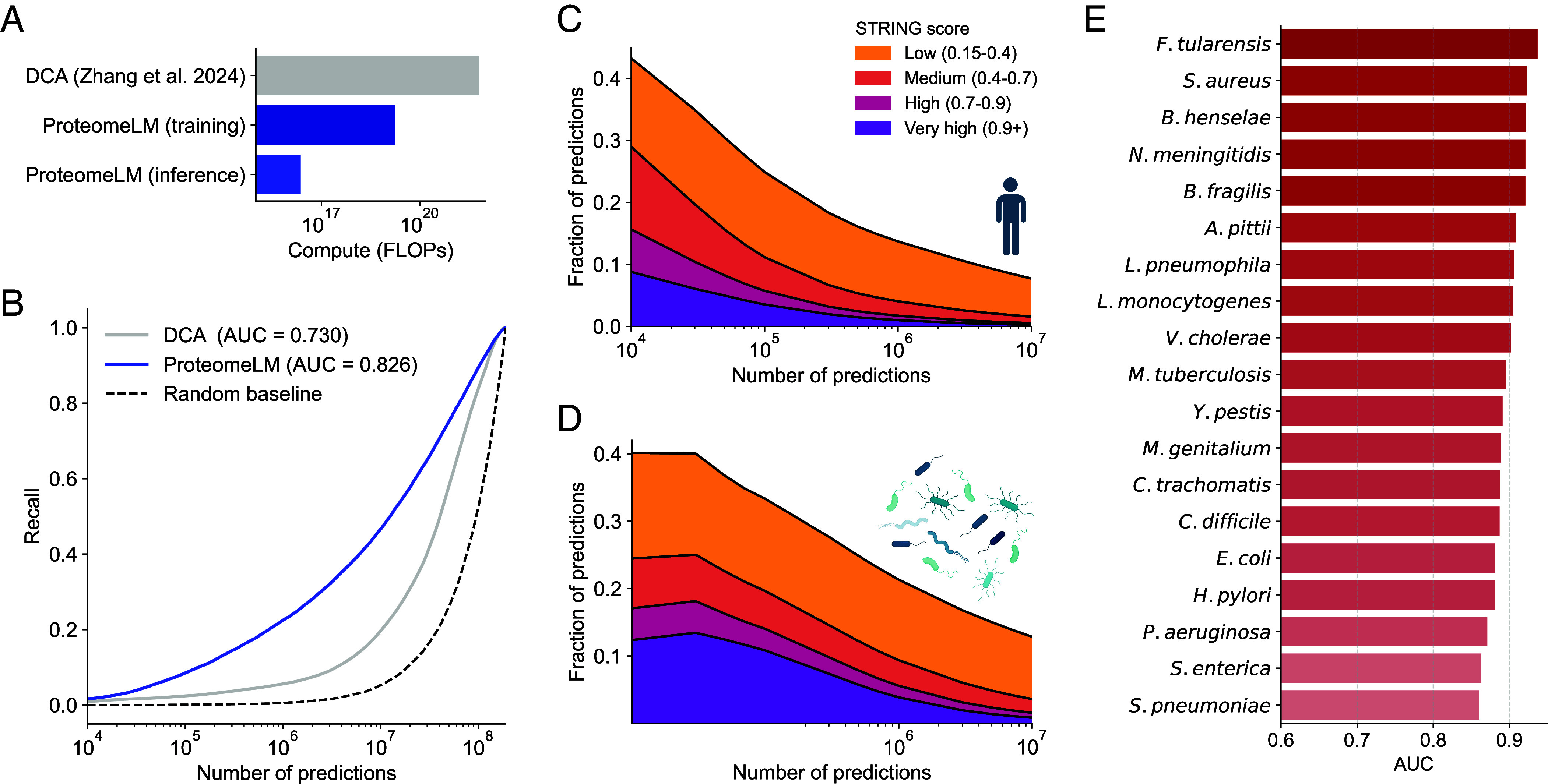
Fast and high-precision screening of whole interactomes using ProteomeLM. (*A*) Compute required to analyze the full human proteome using ProteomeLM vs. DCA. We compare the compute needed to train 190 million DCA models in order to screen the *H. sapiens* proteome (72) with the total compute we used to train ProteomeLM (which was done once across a large number of organisms) and with the compute required to apply ProteomeLM to the *H. sapiens* proteome (inference). ProteomeLM offers a speed-up of up to 6 orders of magnitude when focusing on inference, and of 3 orders of magnitude when including training. (*B*) *H. sapiens* interactome recovery by ProteomeLM and by DCA. Recall is shown vs. the number of predictions made. Predictions are made by ranking all possible protein pairs by their score, either from Proteome or from DCA (72). (*C* and *D*) Fraction of top-scoring predictions that correspond to known interactions in the STRING database, for *H. sapiens* (*C*) and 19 human bacterial pathogens (*D*). (*E*) Interactome recovery performance across 19 pathogenic bacterial species, measured by the AUC.

In ref. 72, the DCA pipeline required over 30 d on 50 to 100 GPUs to process the human proteome. In contrast, ProteomeLM inference, including ESM-C embeddings and attention coefficient computation, takes under 10 min per proteome (e.g., *H. sapiens*) on a single RTX A6000 GPU. Importantly, these features are calculated for all possible protein pairs, without the need to train a separate model for each candidate pair. Moreover, ProteomeLM training was completed in 3 d on a single H100 GPU, and generalizes to all downstream tasks without retraining. As shown in Fig. 3*A*, ProteomeLM thus reduces overall compute time by up to six orders of magnitude for inference alone, and by three orders of magnitude when training is included (see *SI Appendix*, section 4 for details).

Moreover, Fig. 3*B* shows that ProteomeLM significantly outperforms DCA in recovering experimentally validated interactions. In *H. sapiens*, ProteomeLM achieves an AUC of 0.83, compared to 0.73 for DCA. Among the top 10 million scored pairs, it recovers 50% of known PPI, vs. only 20% for DCA. At higher precision thresholds (e.g., top 1 million), ProteomeLM also delivers improved recall. Thus, ProteomeLM has a very strong potential to reduce the burden on downstream structure-based modeling for precise PPI prediction.

We further examine the overlap between ProteomeLM’s top predictions and STRING (69) annotations. Fig. 3*C* shows that in *H. sapiens*, over 40% of the top 10,000 predictions align with known or suspected interactions, and nearly 10% correspond to high-confidence interactions according to STRING. This suggests that ProteomeLM captures meaningful biological associations, even beyond the most confidently labeled pairs.

We extend our analysis to 19 human bacterial pathogens (71). Fig. 3*D* shows that more than 40% of the top 10,000 predictions are supported by STRING. The per-species breakdown is shown in *SI Appendix*, Fig. S9, revealing consistent patterns of STRING support of ProteomeLM predictions across diverse pathogens. Fig. 3*E* further reports AUC values for each of the 19 species, ranging from 0.87 to 0.92. These results confirm that ProteomeLM generalizes well and maintains consistently strong performance across highly diverse taxa.

Together, these findings demonstrate that ProteomeLM is a fast, scalable, and accurate framework for interactome screening. Its ability to recover known interactions and predict plausible novel ones, combined with strongly reduced computational costs, demonstrates its strong potential as a replacement for amino acid coevolution-based filters in proteome-scale PPI prediction pipelines. ProteomeLM therefore opens the way to high-throughput interactome inference across species, including for organisms lacking curated interaction data.

### ProteomeLM Delivers State-of-the-Art Supervised PPI Prediction.

**Fig. 4.**
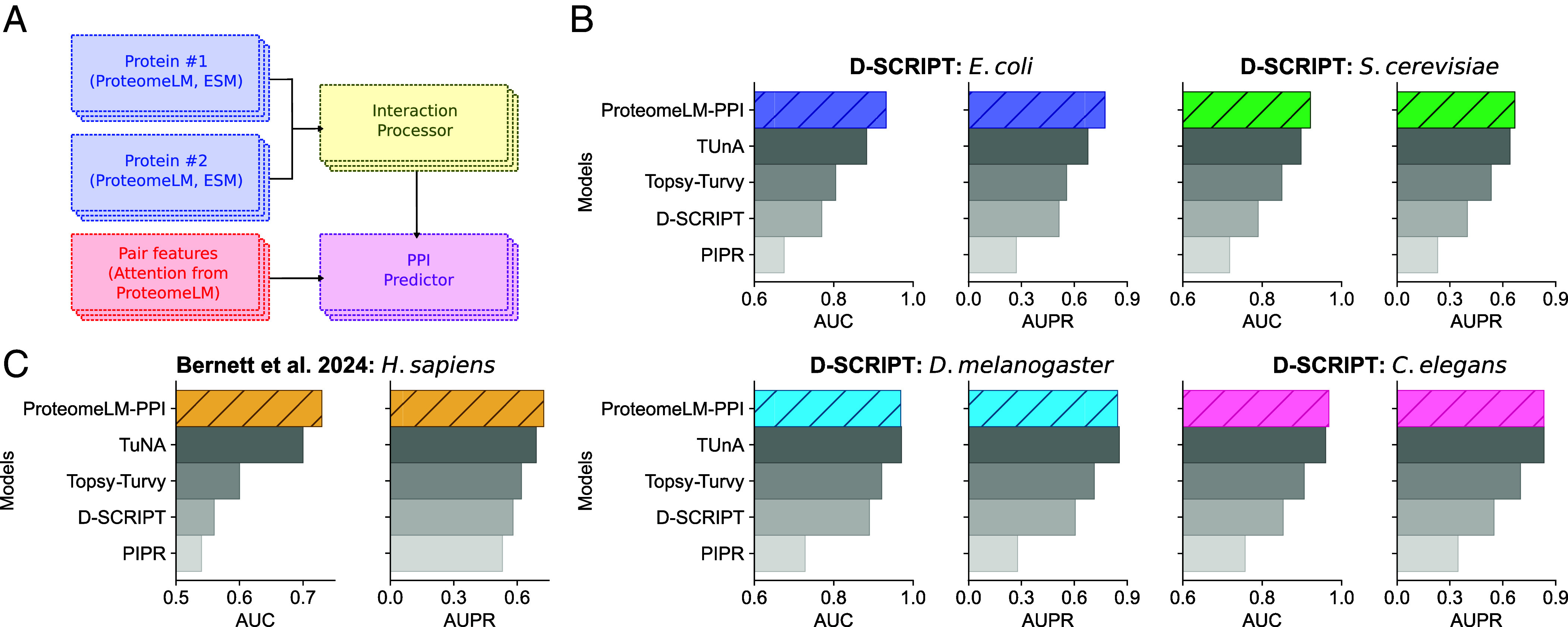
Supervised prediction of PPI using ProteomeLM. (*A*) Architecture of the supervised model trained to predict PPI. The model comprises four components. For each protein in a candidate pair, its representations from ProteomeLM-S and ESM-C are independently processed through a feature encoder (blue), which is a feedforward neural network with two hidden layers. The resulting node features are combined in an interaction processor (yellow) that captures joint information. In parallel, edge features from ProteomeLM attention coefficients are processed through a separate module (red). The final PPI predictor (pink) is a classifier that integrates both node- and edge-level features to predict the interaction probability. (*B*) Cross-species generalization performance on the D-SCRIPT dataset (27), compared to state-of-the-art methods (27, 73–75). All models are trained and validated on human PPI and tested on other species, and results are averaged over five technical replicates. (*C*) Performance on the dataset from ref. 76, averaged over five technical replicates and compared to state-of-the-art methods. In panels *B* and *C*, results are provided in terms of AUC and area under the precision–recall (AUPR) curve.

Since ProteomeLM learns PPI through its attention coefficients without using interaction labels (Fig. 2), and can provide accurate and fast PPI screening, it is tantalizing to further exploit this signal. Can features from ProteomeLM advance supervised sequence-based PPI prediction? To address this question, we introduce ProteomeLM-PPI, a supervised PPI prediction network that employs both node-type features (i.e., embeddings from ESM-C and ProteomeLM for individual proteins) and edge-type features (i.e., ProteomeLM attention values for pairs of proteins), see Fig. 4*A*.

We trained and evaluated ProteomeLM-PPI on two PPI datasets. The first one is the multispecies D-SCRIPT dataset (27), already used in the previous sections. The second one is a human-specific dataset which was designed to address common biases in the training, validation, and test splits of previous PPI benchmarks, in particular leakage (76). For both datasets, we used the same training, validation, and test splits as in the recent TUnA method (75). ProteomeLM-PPI was trained on the training set, with early stopping based on validation performance to avoid overfitting.

In the D-SCRIPT dataset, the training set is composed of human PPI and the validation set comprises held-out human PPI (75). Testing on PPI from other species thus allows to assess cross-species generalization power. Fig. 4*B* shows that ProteomeLM-PPI outperforms state-of-the-art methods on *E. coli* and *S. cerevisiae*, and performs comparably to them on *D. melanogaster* and *C. elegans*. In particular, it leads to an AUPR improvement of more than 0.1 (from 0.67 to 0.79) over the previous state of the art, TUnA (75), on *E. coli*. These results highlight ProteomeLM’s strong ability to capture PPI signals, its robustness across diverse branches of the tree of life, and its ability to generalize from one species to other ones.

In Fig. 4*C*, we report results on the dataset from ref. 76. We observe that ProteomeLM-PPI yields very strong AUC and AUPR scores, consistently reaching or outperforming state-of-the-art methods including TUnA, which employed ESM-2 embeddings. This result shows the robustness of ProteomeLM-PPI to biases of PPI prediction benchmarks (76).

To better understand the contribution of each feature used by ProteomeLM-PPI, we evaluate different combinations of these features in *SI Appendix*, Fig. S10. We observe that both ProteomeLM embeddings and attention coefficients are individually informative, and that their combination consistently yields the best predictive performance. This suggests that embeddings and attention coefficients contain complementary information that is synergistic for PPI prediction.

### ProteomeLM Improves Gene Essentiality Prediction.

While we focused on PPI prediction so far, ProteomeLM is a foundation model that can be used for diverse tasks where proteome-level information is important. Here, we consider another important task, which consists in predicting which genes are essential, i.e., necessary for survival or reproduction of an organism. Both protein or gene sequence on the one hand, and genomic context and PPI on the other hand, have been found to matter for predicting essentiality (62). Can ProteomeLM, which exploits these diverse elements, advance gene essentiality prediction?

To address this question, we introduce ProteomeLM-Ess, a supervised predictor of gene essentiality that takes as input ProteomeLM embeddings. To train and test this classification model, we used the OGEE database (77), which collects gene essentiality data from 127 experimental studies, spanning 91 species, for a total of 213,608 labeled genes. To create the training, validation and test split, we clustered proteins based on sequence similarity (*Materials and Methods*). ProteomeLM-Ess is a two-layer fully connected network, and can take as input ProteomeLM embeddings from any layer.

**Fig. 5.**
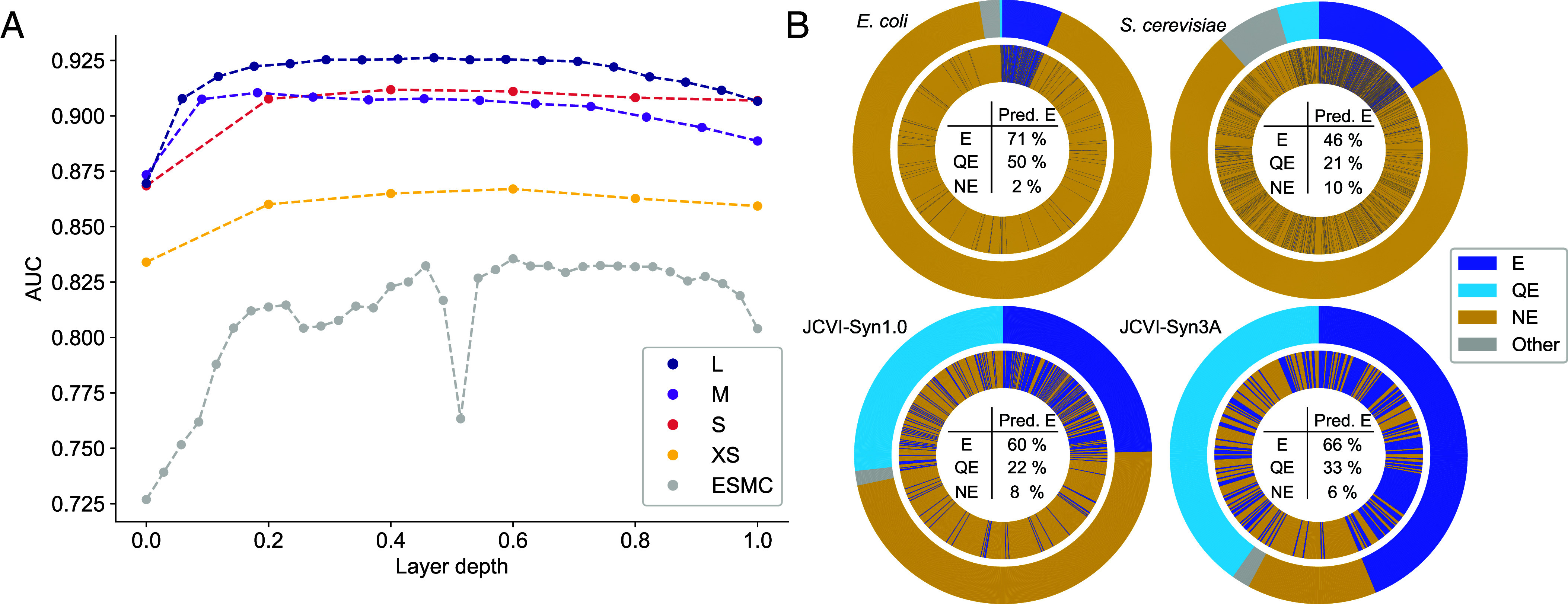
Gene essentiality prediction with ProteomeLM-Ess. (*A*) Area under the ROC curve (AUC) for binary classifiers that take as input embeddings from each layer of ProteomeLM (ProteomeLM-Ess) or ESM-C. To facilitate comparison between models with different sizes, the AUC is plotted vs. the normalized layer depth, i.e., the ratio of the layer index to the number of layers. (*B*) Comparison between ProteomeLM-Ess predictions (inner rings) and experimental essentiality labels (outer rings) for *E. coli* K-12, *S. cerevisiae*, and the synthetic cells JCVI-Syn1.0 and JCVI-Syn3A. ProteomeLM-Ess uses embeddings from layer 8 of ProteomeLM-L. These genomes are not in the training set of ProteomeLM-Ess. For each of them, we predict as essential the top N genes in terms of ProteomeLM-Ess score, where N is the number of genes experimentally labeled essential. While ProteomeLM-Ess classifies genes as essential (E) or nonessential (NE), some genes in these species (many in minimal cells) are labeled as quasi-essential (QE). The percentage of genes labeled as E, QE, or NE that are predicted as E by ProteomeLM-Ess is indicated in each case. AUC values: *E. coli*, 0.95; *S. cerevisiae*, 0.81; JCVI-Syn1.0, 0.88; JCVI-Syn3A, 0.83.

Fig. 5*A* shows the performance of ProteomeLM-Ess vs. the depth of the layer whose embeddings are used as its input, for all ProteomeLM sizes. This performance is also compared to that of a similar classifier that starts from ESM-C embeddings instead of ProteomeLM ones. We observe that classifiers based on ProteomeLM embeddings significantly outperform those based on ESM-C embeddings. This demonstrates that the contextualized whole proteome-aware information present in ProteomeLM embeddings allows to better capture gene essentiality than protein-level information. Besides, we observe that the embeddings from intermediate layers of ProteomeLM produce the best gene essentiality classifiers, which is consistent with our observations for unsupervised PPI prediction (Fig. 2*D*). Furthermore, the performance of ProteomeLM-Ess scales with ProteomeLM size. This suggests that larger models are able to encode more information about gene essentiality into their representations. Interestingly, larger sizes appear to be more useful for gene essentiality than for PPI prediction (*SI Appendix*, Fig. S9). Overall, the best performing version of ProteomeLM-Ess is the one trained on the embeddings of layer 8 of ProteomeLM-L, yielding an AUC of 0.93.

Can ProteomeLM-Ess generalize to unseen proteomes? To address this question, we held out the entire proteomes of *S. cerevisiae* and of *E. coli* during training. Fig. 5*B*, *Top*, shows the performance of ProteomeLM-Ess on these two held-out proteomes. We obtain good results, especially in *E. coli*, where 71% (resp., 2%) of genes experimentally determined to be essential (resp., nonessential) were correctly predicted as essential (resp. nonessential) by ProteomeLM-Ess. To further explore generalization ability, we collected essentiality data for the synthetic cells JCVI-Syn1.0 (78) and JCVI-Syn3A (79, 80), which are not present in OGEE. Moreover, JCVI-Syn3A has a genome that is engineered to be close to minimal, so it is both far from genomes in the training set and a stringent test of gene essentiality prediction. Fig. 5*B*, *Bottom*, shows that we obtain good performance in these cases too, even for JCVI-Syn3A. Hence, ProteomeLM-Ess is able to generalize to unseen taxa.

We further compare ProteomeLM-Ess to several existing gene essentiality prediction methods (24, 25, 81–88) on *E. coli* and *S. cerevisiae*, which were both held out from the training set. Overall, ProteomeLM-Ess achieves state of the art performance for *E. coli*. It is also state of the art for *S. cerevisiae* among methods trained without labeled data from that species. Methods trained using labeled data from *S. cerevisiae*, however, perform as well as or better than ProteomeLM-Ess (*SI Appendix*, section 5).

## Discussion

We introduced ProteomeLM, a transformer-based language model that learns contextualized protein representations from complete proteomes spanning the whole tree of life. Trained to reconstruct masked protein representations from the other proteins of a proteome, ProteomeLM learns dependencies between proteins that reflect functional and evolutionary constraints. This makes it well-suited to predict proteome-scale properties. We showed that ProteomeLM captures PPI in its attention heads, without any supervision. Specifically, ProteomeLM is an excellent predictor of both broad functional relationships and protein complex membership, and can distinguish them.

We then demonstrated that ProteomeLM can be used as a highly scalable and effective tool for whole-interactome screening, yielding substantially higher accuracy than DCA, with a computational cost reduced by up to 6 orders of magnitude. This makes ProteomeLM a strong choice for large-scale PPI screening tasks, including in poorly annotated organisms. Next, we designed ProteomeLM-PPI, a supervised PPI prediction network that leverages both embeddings and attention coefficients from ProteomeLM, and achieves state-of-the-art performance across species and benchmarks. Finally, we trained ProteomeLM-Ess, a supervised classifier for gene essentiality prediction that starts from ProteomeLM embeddings. We showed that classifiers trained on ProteomeLM embeddings outperform those trained on ESM-C embeddings for this task, confirming that ProteomeLM’s contextualized embeddings encode biologically relevant proteome-scale information.

In summary, ProteomeLM is a framework that learns whole proteome-aware protein representations across the tree of life. As a foundation language model trained using the MLM objective, ProteomeLM can be used for many downstream tasks. The possibility of using the pretrained ProteomeLM, and even of employing precomputed ProteomeLM embeddings, means that using it in inference for downstream tasks can be very computationally efficient. We demonstrated that ProteomeLM reaches very strong performance on tasks ranging from PPI screening to gene essentiality prediction, and combines speed, interpretability, and accuracy. We expect that proteome-aware language models will become increasingly important in the coming years, enabling new ways of modeling system-level biological properties at the scale of proteomes and cells.

Integrating local genomic context to protein language model embeddings has proved very useful for function inference(22, 65, 89) and genome annotation (90). ProteomeLM demonstrates the power of integrating whole-proteome information across species. ProteomeLM reasons on proteomes at a coarse-grained level, representing each protein by a global representation from the protein language model ESM-C. These light representations allowed us to maintain reasonable context sizes and to work with full proteomes. In the future, the progress of long-context language models may enable ProteomeLM to directly operate at the amino acid level across whole proteomes, paving the way to localized cross-protein interactions and more granular modeling of functional dependencies. One could even envision using full genomic nucleotide sequences, in the spirit of recent models that work on local genomic neighborhoods (17–19, 24–26). While this may become possible, we expect models working on complete raw genomic sequences to be much heavier and to require much more training data, as they need, e.g., to learn about coding regions and codons. Note that alternative architectures could help address longer sequences, as for protein language models (91, 92). Models operating on raw genomic sequences can address regulatory functions or the role of noncoding DNA (17–19), which is beyond the scope of ProteomeLM. However, once they reach whole genomes, it remains to be seen whether they can efficiently and accurately predict proteome-level tasks such as PPI and gene essentiality, where ProteomeLM excels.

A major asset of ProteomeLM, allowed by our introduction of functional encodings instead of positional encodings, is that it can reason on proteomes across the tree of life. Nevertheless, in terms of absolute scores, ProteomeLM’s performance remains stronger on prokaryotes than on eukaryotes. This may be due to the relative scarcity of high-quality eukaryotic proteomes in the training dataset and to the higher complexity of eukaryotic genome organization and gene regulation. While fine-tuning ProteomeLM on eukaryotic-only data, or training it only on that data, brings minor improvements on eukaryotes, it comes at the cost of reduced performance on bacteria. As eukaryotic genomic databases continue to grow, larger training sets may allow us to overcome this limitation. Along these lines, expanding the training set to include more eukaryotic proteomes, including less-well annotated ones, and metagenomic proteomes, should further improve ProteomeLM’s performance across taxonomic groups.

Another interesting perspective for future work is to train ProteomeLM using embeddings from protein language models trained over additional modalities, beyond sequences, such as structure [SaProt (93), ProstT5 (94), ESM-3 (95), ProtTrek (96)]. Exploiting the complementary information available in structure and sequences (97) may help ProteomeLM infer more complex functional relationships. This direction is particularly promising for PPI prediction, as shown by recent work (27, 98–102).

ProteomeLM opens the way to several applications. Given its computational efficiency at inference, it can be used to map functional networks and coexpression clusters, predict protein complex membership across species, and study the evolution of these systems. For the determination of direct physical interactions, it can serve as a fast and accurate screening step to prioritize candidate protein pairs for computationally intensive analyses, including structural prediction approaches such as Boltz (103, 104) and AlphaFold3 (105), or interface prediction frameworks such as PIONEER 2.0 (102). Beyond interaction prediction, ProteomeLM enables high-throughput in silico screens of gene essentiality and comparisons across species, and could be extended to investigate joint essentiality (106–110) and environment-dependent essentiality (111). ProteomeLM embeddings can further serve as a basis for other downstream tasks such as context-aware fitness prediction, opening the possibility of large-scale investigations of fitness landscapes.

## Materials and Methods

### Architecture and Training of ProteomeLM.

#### Dataset.

We collected 31,947 proteomes from OrthoDB (version 12) (67). Each of these proteomes is a list of protein sequences annotated by their respective orthologous groups. An orthologous group comprises descendants of a common ancestral gene, separated by speciation, and usually retaining the same function. It is linked to functional annotations from gene ontology (GO) (112, 113), describing protein localization and biological processes. However, here, we do not use these functional annotations. Our functional encoding (described below) only relies on orthologous groups. Note that the definition of an orthologous group operates at a specific level of orthology. Here, we use all these levels (see below).

#### Protein representations.

We used the ESM-Cambrian (ESM-C) model with 600 million parameters (12) to represent each of the 162 million proteins in our dataset. ESM-C is a protein language model, trained on a vast corpus of sequences, which is a successor of ESM-2 (10). Note that, contrary to ESM-3, which is multimodal (95), it is a sequence-only model. We selected ESM-C because of its strong performance in capturing the structural and functional properties of protein sequences. Each protein sequence was encoded by ESM-C, and the per-amino acid ESM-C embeddings were averaged to obtain a global embedding of the protein with a fixed dimension of 1152.

To optimize computational efficiency, protein sequences were batched based on their length, reducing the overhead associated with the model’s quadratic time complexity. The whole embedding computation process took 192 GPU-hours on one H100 GPU.

#### Functional encoding.

In natural language processing (114), and also for protein sequences (9, 12, 115) and for genomic sequences (18, 22, 89), BERT models usually rely on positional encoding, which provides the model with information on the order of tokens (words in a sentence, amino acids in a protein chain, nucleotides along the genome). However, such a positional encoding is not appropriate for our purpose, given the lack of conservation of genomic order across diverse species, and the lack of correlation between proximity along the genome and functional relationship or interaction in eukaryotic genomes. Instead, we designed a functional encoding based on OrthoDB orthologous groups, which provides the model with information about the functional identity of each protein.

OrthoDB organizes orthologous groups in a tree that mirrors the taxonomy of life. At the finest level, a leaf orthologous group contains a set of orthologous proteins from closely related species (e.g., Hominidae). Each leaf group is nested within progressively broader groups defined at higher taxonomic levels (e.g., Mammalia, Vertebrata, Metazoa), up to a root encompassing all cellular organisms. Moving up this hierarchy, orthologous groups become more inclusive, reflecting more ancient and broadly conserved functions. We exploit this hierarchy to construct a multiscale functional representation for each protein, as follows. First, for each leaf orthologous group, containing individual proteins but no subgroups, we average the ESM-C embeddings of all proteins in that group, yielding a vector characterizing that specific orthologous group. Second, we propagate these representations through the hierarchy. Going from leaves to root, we compute a vector for each parent group as the equal-weight average of its immediate children’s vectors. Having each child orthologous group contribute equally to the parent representation prevents large groups from dominating. Thus, every node in the OrthoDB hierarchy is assigned a vector. For each protein, we collect all vectors representing each group along the ancestral path from its leaf orthologous group to the root. This yields a set of vectors that describe the protein’s functional identity at each taxonomic level. Finally, for proteins which are not members of an OrthoDB group, we use their ESM-C embedding as their functional encoding.

During training, for each protein in a proteome, the functional encoding is obtained by randomly sampling (at each epoch) one vector from its ancestral path. This stochastic sampling exposes the model to functional descriptions at varying levels of specificity during training, encouraging it to learn relationships between proteins that hold at multiple evolutionary scales.

We find that these functional encodings outperform simpler ones, based on a discrete representation of orthologous group identities at the broadest taxonomic level (*SI Appendix*, section 6). Our use of hierarchically averaged ESM-C embeddings captures richer signal, especially for predicting physical PPI.

#### Architecture.

We trained a transformer encoder from scratch to learn complex relationships between the ESM-C embeddings of different proteins in a proteome. The core of the model is the DistillBERT architecture, available in Hugging Face’s transformers library (116). We used FlashAttention-2 (117) to accelerate training and inference.

Each proteome is represented as a list of protein embeddings with their associated functional encodings. For each protein, the ESM-C embedding and the functional encoding are given as inputs to ProteomeLM, through two separate linear projection layers.

#### Training objective and loss design.

ProteomeLM is trained using a masked language modeling (MLM) objective adapted to proteome-level inputs. During the training of ProteomeLM, we limit the size of the proteomes to 4096 by randomly subsampling proteins when proteomes are longer. While longer inputs could in principle be employed, since we use FlashAttention, which has linear memory complexity, we chose to limit the length of the input to 4096 to reduce computational time, which still has quadratic complexity. We randomly mask 50% of the protein representations within a proteome, while their functional encodings are kept unmasked. Masked proteins are replaced by their functional encoding, and the model is trained to reconstruct the original protein embeddings based on contextual signals from the rest of the proteome.

The standard masked language modeling loss cannot be applied here, because we work directly with continuous embeddings and not with discrete tokens. A straightforward alternative would be the mean squared error (MSE) between the actual embedding x and the predicted embedding $\hat{x}$. However, this approach results in a degenerate solution where the model simply reproduces the functional encoding $\overset{¯}{x}$. Indeed, when expressed in terms of the residuals $r=x-\overset{¯}{x}$ and $\hat{r}=\hat{x}-\overset{¯}{x}$, the MSE becomes insensitive to the angle θ between r and $\hat{r}$ when x approaches $\overset{¯}{x}$, and thus fails to penalize incorrect residual directions (*SI Appendix*, section 7). To address this challenge, we decouple the residual magnitude and direction. We formulate the residual prediction problem probabilistically under this decoupling, using tractable assumptions on the distributions of residual magnitudes and angles. A maximum-likelihood derivation under this model leads to the following custom polar loss (*SI Appendix*, section 7):

[1] $$\begin{array}{c} L_{\text{Polar}}\left(\hat{x},x,\overset{¯}{x}\right)=\left(1-\cos\theta \right)+{\left(\|\hat{r}\left\|-\|r\right\|\right)}^{2}\;. \end{array}$$

The first term corresponds to the cosine embedding loss applied to the residuals, and encourages alignment between r and $\hat{r}$. The second term penalizes discrepancies in residual magnitude through the squared difference between the Euclidean norms of r and $\hat{r}$. The polar loss is minimized if and only if $\hat{x}=x$, thereby avoiding the failure mode of the MSE loss. Empirical comparisons are given below in *Comparison of Losses*.

#### Training dynamics and scaling behavior.

We trained four variants of ProteomeLM, differing by model sizes: XS (5.6M parameters), S (36M), M (112M), and L (328M). All models were trained for 210 epochs on a dataset comprising 31,000 proteomes with a total of 160 million proteins. Validation loss was measured on a 2% held-out set of proteomes randomly sampled from the training set.

**Fig. 6.**
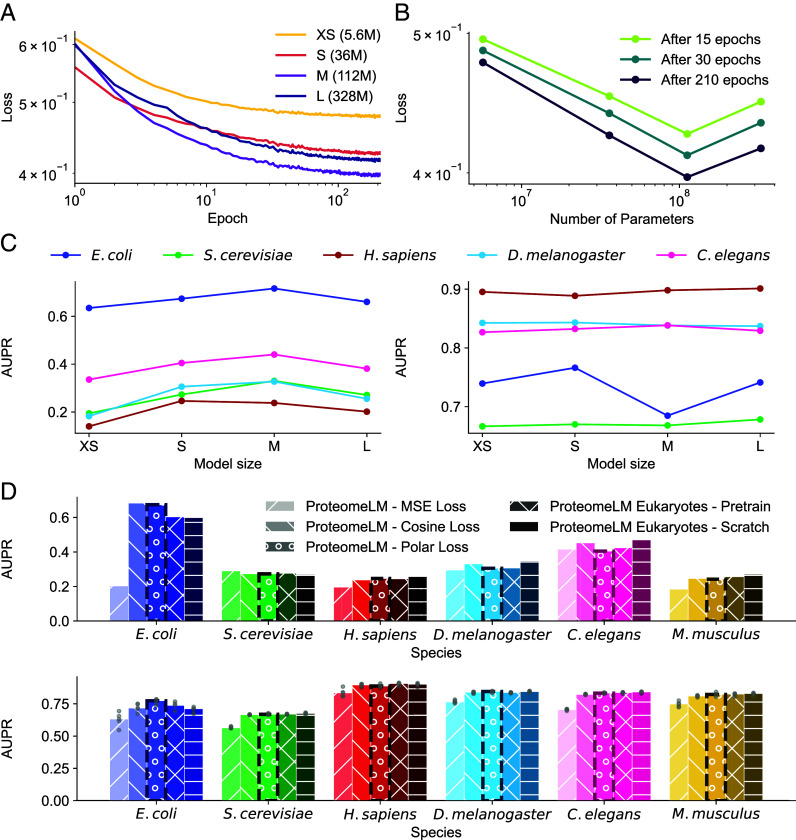
Training dynamics, scaling behavior, and training strategies for ProteomeLM. (*A*) Evaluation loss during training for four ProteomeLM model sizes: XS (5.6M parameters), S (36M), M (112M), and L (328M). (*B*) The final evaluation loss is shown for three different epochs. (*C*) The AUPR for unsupervised PPI prediction using summed attention coefficients over all heads and layers (*Left*) and supervised PPI prediction through the ProteomeLM-PPI architecture (*Right*) is shown across five species on the D-SCRIPT dataset. (*D*) Comparison of three different loss functions (MSE, cosine, and polar, where polar is the one retained throughout), and evaluation of two training strategies focused on eukaryotic data (fine-tuning and training from scratch). AUPR are shown for both unsupervised (*Top*) and supervised (*Bottom*) PPI prediction tasks. As in *C* (*Left* panel), summed attention coefficients over all heads and layers are used for unsupervised prediction. (*C* and *D*) All models used in these comparisons were trained for 210 epochs under identical hardware and random seed conditions to ensure fair evaluation.

Training remained stable across all model sizes, showing smooth convergence, see Fig. 6*A*. Fig. 6 *A* and *B* show that performance, assessed by loss value, improved steadily from XS to M, suggesting that the model benefits from increased capacity. However, the L model failed to outperform M, and in some cases showed degraded performance. In particular, in Fig. 6*B*, the trend follows a scaling law from XS to M, before performance degrades for the L model. We attribute this to overfitting, given that the number of trainable parameters exceeds the number of unique training proteins in the training set for ProteomeLM-L. To rule out architecture-specific factors, we tested variants of ProteomeLM-L with different numbers of layers, heads, and embedding dimensions. These variants exhibited similar behavior in early training, reinforcing the interpretation that training data volume is the limiting factor.

In *SI Appendix*, Fig. S11, we show the training dynamics of each attention head in ProteomeLM-S by tracking their AUC for unsupervised PPI recovery during training. Certain heads become increasingly predictive of PPI as training progresses. Some of them display taxon-specific specialization, while others exhibit consistent predictive power across species. These results highlight that ProteomeLM’s attention heads learn distinct, biologically meaningful signals during training.

We also used the D-SCRIPT dataset (27) to assess the performance of the models on PPI recovery (training and validation on disjoint sets of *H. sapiens* PPI, test on other species). The *Left* panel of Fig. 6*C* shows that, for unsupervised PPI recovery, AUPR increases with model size up to M, and decreases for size L, thus confirming the trend observed for the loss. Note also that performance on human data slightly decreased from S to M, indicating that larger models do not always generalize better across all species. The *Right* panel of Fig. 6*C* shows that AUPR increases over model size on eukaryotes, while it degrades after model S on *E. coli*, again showing the better generalization capabilities of the smaller models.

#### Comparison of losses.

We evaluated our polar loss function against two natural alternatives, namely the MSE loss between $\hat{x}$ and x and the cosine embedding loss between $\hat{x}-\overset{¯}{x}$ and $x-\overset{¯}{x}$. (Note that we do not consider the cosine embedding loss between $\hat{x}$ and x because these vectors tend to have a similar orientation anyway within each protein family.) For our comparison of loss functions, we trained a version of ProteomeLM for 72 h with each of these two alternative losses. Performance comparisons were conducted in both unsupervised and supervised PPI prediction tasks. In the unsupervised setting, we used the mean of attention weights over all heads and layers as a simple estimate of interaction probability for each protein pair. In the supervised setting, we trained a downstream classifier (Fig. 4*A*) using frozen representations obtained from models trained with each loss function.

As shown in Fig. 6*D*, our polar loss consistently outperformed both the MSE loss and the cosine loss across multiple evaluation metrics. In particular, the unsupervised AUC scores were generally performing higher when the model was trained with the polar loss or the cosine embedding loss than with the MSE loss. Likewise, in the supervised PPI prediction task, the models trained with the polar loss yielded higher precision–recall performance (AUPR). The MSE loss showed the weakest performance in both settings. As mentioned above, it is because the model then converges toward a degenerate solution, where the model simply reproduced the functional encoding ($\hat{x}=\overset{¯}{x}$).

Note that, in addition to predictive accuracy, we observed that the polar loss produced embeddings with properties more closely aligned to those of ESM-C, thus facilitating interoperability between models, e.g., improving compatibility in transfer learning applications. These results validate the polar loss as an appropriate objective for reconstructing protein embeddings within a proteome context.

#### Improving performance on eukaryotic data.

We observe that ProteomeLM’s accuracy is comparatively lower on eukaryotic datasets than on prokaryotic ones (Figs. 2 and 4). We explored two approaches to improve performance on eukaryotes: fine-tuning the pretrained ProteomeLM on eukaryotic data and training a new model from scratch on eukaryotic data only.

Fig. 6*D* shows that both approaches provide moderate improvements on eukaryotic benchmarks, both for unsupervised and supervised PPI prediction. However, these improvements come with a decrease in performance on prokaryotes such as *E. coli*, indicating a trade-off between specialization and generalization.

Given our goal to design a model that works across diverse organisms, we retained the baseline ProteomeLM models for our main analyses. However, the specialized eukaryotic alternatives can be valuable for specific applications.

### Supervised PPI prediction: ProteomeLM-PPI.

#### Architecture and input.

The supervised ProteomeLM-PPI model relies on a modular neural network that processes both individual protein embeddings (node features) and attention coefficients (edge features) through distinct but integrated modules, see Fig. 4*A*.

Specifically, the node feature module reduces each protein embedding from 640 to 256 dimensions through two layers (640 → 512 → 256), with layer normalization and dropout to enhance stability and weight regularization. To model the interaction between two proteins, the network combines their transformed representations by concatenating each of the two representations, their element-wise multiplication, and their absolute difference, thus resulting in a 1024-dimensional vector (4 × 256), which is then compressed to 64 dimensions through the interaction processor (1024 → 128 → 64). In parallel, the edge feature module reduces the 48-dimensional input (from the 48 attention heads of ProteomeLM-S) to 32 dimensions (48 → 64 → 32). The 64-dimensional processed interaction features from the interaction processor are then concatenated with the 32-dimensional pairwise feature vector to form a 96-dimensional input to the final PPI prediction classifier module. This input then passes through two layers (dimensions: 96 → 128 → 64), before the PPI predictor outputs a final interaction score. This modular design enables the model to flexibly integrate different sets of learned features while maintaining strong inductive biases for capturing protein–protein relationships.

#### Training.

To train the model, we used a train–validation–test split. During training, the model is optimized on the training set, while its performance is monitored on the validation set to apply early stopping, preventing overfitting. During training, the protein pairs of the training set are fed into the model in minibatches as triplets comprising ProteomeLM embeddings of both proteins involved, and ProteomeLM attention weights between the two of them. The training minimizes binary cross-entropy with logits using the Adam optimizer (118). At each epoch, predictions on the validation set are evaluated using the AUPR curve, and the best model state is saved based on this metric. After training, the best model is evaluated on the held-out test set, and we report AUC and AUPR.

Our training approach ensures a fair assessment of the model’s generalization ability by performing model selection on a dedicated validation set, rather than the test set, thereby avoiding overfitting to the test data. For both the dataset from ref. 76 and the D-SCRIPT dataset (27), clean and nonoverlapping splits into training, validation, and test sets were already available, and we employed them.

### Supervised Gene Essentiality Prediction: ProteomeLM-Ess.

#### Architecture and input.

ProteomeLM-Ess is a two-layer fully connected classifier that takes as input embeddings from any ProteomeLM model, has a hidden layer of size 2048, and outputs two logits, which are normalized with a softmax function to obtain an essentiality score. In the hidden layer, ProteomeLM-Ess has a ReLU activation and dropout with probability 0.5. Protein embeddings are normalized using the genome-wide mean and SD before being given as input to ProteomeLM-Ess.

#### Data and training.

ProteomeLM-Ess is trained in a supervised way, using ProteomeLM embeddings together with essentiality labels from the OGEE database (77). We recovered the protein sequences associated to the essentiality labels from other databases, by matching the gene names (gene IDs) provided by OGEE. Specifically, we collected protein sequences from UniProt (119), NCBI (120), *Saccharomyces* Genome Database (SGD) (121) and Fitness Browser (122), and obtained data for 87 taxonomic IDs. Relying on curated complete proteomes whenever possible allowed to minimize ambiguities coming from the presence of isoforms or duplicate protein sequences (i.e., identical sequences with different protein IDs). The remaining duplicate entries were merged, while keeping both IDs.

Out of the 87 total genomes, we used 83 to train ProteomeLM-Ess, holding out the genomes of *S. cerevisiae* and of four strains of *E. coli*. We also collected essentiality data for the synthetic cells JCVI-Syn1.0 (78) and JCVI-Syn3A (79, 80), to evaluate the model after training. For the 83 genomes used for training, we split the proteins into training, validation, and test sets by clustering proteins across all genomes according to sequence similarity. Specifically, we clustered sequences using MMSeqs2 (123) with a 40% similarity threshold. We designed our split so that if two labeled proteins belong to the same cluster, then they are either both in the training set, in the validation set, or in the test set. The data split is performed at the protein level and not at the genome level, to avoid the model relying on sequence similarity between, say, two orthologs in similar genomes, as a shortcut to predict essentiality. All protein sequences are given as input to ProteomeLM to build contextualized embeddings. The training procedure and objective used for ProteomeLM-Ess are the same as the ones used for ProteomeLM-PPI (see above).

## Supplementary Material

Appendix 01 (PDF)

## Data Availability

We provide code for training and using ProteomeLM at https://github.com/Bitbol-Lab/ProteomeLM (124). Code weights, and all the material needed to reproduce our results, are available there.
