# Supplementary material for: ProteomeLM: A proteome-scale language model enables accurate and rapid prediction of protein–protein interactions and gene essentiality across taxa
Source: Proc Natl Acad Sci U S A. 2026 May 20;123(21):e2524201123. doi: 10.1073/pnas.2524201123 (PMC13214046; doi:10.1073/pnas.2524201123)
Supplement: Supplementary file 1 — Appendix 01 (PDF) [file pnas.2524201123.sapp.pdf]

Supplementary Information for "ProteomeLM: A proteome-scale language model enables accurate and rapid prediction of protein-protein interactions and gene essentiality across taxa"

Cyril Malbranke[1,2,*], Gionata Paolo Zalaffi[1,2], Anne-Florence Bitbol[1,2,*]

1 Institute of Bioengineering, School of Life Sciences, EPFL, CH-1015 Lausanne, Switzerland
2 SIB Swiss Institute of Bioinformatics, CH-1015 Lausanne, Switzerland
* Corresponding authors: `cyril.malbranke@epfl.ch`, `anne-florence.bitbol@epfl.ch`

April 23, 2026

Contents

1 Distinguishing physical interactions from functional associations 2

2 Application of ProteomeLM to structurally resolved protein complexes 3
2.1 *E. coli* ribosome . . . 3
2.2 *S. cerevisiae* TRiC/CCT chaperonin . . . 4

3 Improvement of learned representations over input representations 6
3.1 Cosine similarity of input embeddings and functional encodings . . . 6
3.2 PCA removal from the input similarity matrix . . . 7
3.3 Supervised evaluation with sequence-similarity-controlled splits . . . 7

4 Comparison of compute requirements for human interactome scanning 8

5 Comparison of ProteomeLM-Ess with other gene essentiality prediction methods 9

6 Comparison of continuous and discrete functional encodings 11

7 Theoretical motivation of the polar loss used to train ProteomeLM 12

8 Supplementary figures 14

1 Distinguishing physical interactions from functional associations

ProteomeLM learns statistical dependencies between proteins based on their context across thousands of genomes, and shares similarities with phylogenetic profiling [1]. Do ProteomeLM's attention coefficients capture direct physical binding, broader functional associations, or both? To address this, we compare ProteomeLM attention coefficients across different types of protein-protein associations.

Benchmark construction. For *E. coli*, *S. cerevisiae*, and *H. sapiens*, we constructed a benchmark comprising the following four categories of protein pairs:

- **Direct interactions (PDB):** Protein pairs involved in the same PDB complex, with buried surface area above 500 $Å^2$ and at least 10 pairs of residues with distance between closest atoms below 8 Å.
- **Same complex interactions (PDB):** Protein pairs involved in the same PDB complex but that do not satisfy the criteria stated above.
- **Coexpression (STRING):** Protein pairs with high coexpression scores (≥ 0.7) in the STRING database [2], representing proteins with correlated expression patterns across experimental conditions.
- **Random pairs:** Randomly sampled protein pairs from the same proteome, serving as negative controls. We ensured that every protein in these pairs is involved in known interactions (from the three previous sets).

Pairs were filtered to ensure no overlap between categories, and random pairs were sampled to exclude any pair present in STRING with non-zero scores.

Results. We first evaluate the strength of the signal in ProteomeLM attention coefficients for each interaction type. Table S1 shows the Area Under the Receiver Operating Characteristic curve (AUROC) for discriminating each interaction type from random pairs, using the mean of ProteomeLM attention coefficients from all heads and layers. Across all three species, coexpression pairs yield the strongest attention signals, achieving AUROC values of 0.92-0.95. Direct and physical interactions are also well detected (0.75-0.92), but with a lower strength than coexpression. Hence, ProteomeLM is a powerful predictor of functional associations and gene co-regulation.

Table S1: **Discriminating three interaction types from random pairs.** All results are given in terms of AUROC. Higher values indicate a stronger attention signal relative to a random background.

Species	Direct	Complex	Coexpression
E. coli	0.873	0.916	0.924
S. cerevisiae	0.810	0.828	0.946
H. sapiens	0.745	0.787	0.946

Next, to assess whether ProteomeLM can distinguish physical binding from broader functional associations, we train logistic regression classifiers to discriminate between interaction types based on attention coefficients. Table S2 demonstrates that coexpression can be well distinguished from direct or same-complex interactions using ProteomeLM. For instance, the classification accuracy when distinguishing direct interactions from coexpression has a 0.89 AUROC in *S. cerevisiae*. However, the more subtle distinction between direct and same-complex interactions is less well captured by ProteomeLM.

Interpretation. These results demonstrate that ProteomeLM is an excellent predictor of broad functional relationships, and also captures specific information regarding physical binding. Furthermore, the attention signatures for physical and genetic interactions are distinct. This suggests a dual utility: ProteomeLM can be used to map functional networks and co-expression clusters, while simultaneously serving as a high-recall filter for structural PPI prediction pipelines by identifying pairs that are physically interacting.

Table S2: **Pairwise binary classification between interaction types.** Ability of logistic regression classifiers trained on ProteomeLM attention heads to distinguish between specific interaction types. All results are given in terms of AUROC.

Species	Direct vs. complex	Direct vs. coexpression	Complex vs. coexpression
E. coli	0.504	0.728	0.626
S. cerevisiae	0.552	0.885	0.851
H. sapiens	0.601	0.904	0.882

2 Application of ProteomeLM to structurally resolved protein complexes

2.1 *E. coli* ribosome

To assess ProteomeLM's ability to recover physical protein interactions within a multi-protein complex, we consider the *E. coli* 70S ribosome, focusing on ground-truth inter-chain contacts extracted from a high-resolution cryo-EM structure (PDB: 7K00, 2.0 Å resolution) [3]. This complex comprises 46 proteins, and provides a benchmark involving a dense network of both direct and indirect PPI within a complex.

Ground truth extraction. We parsed the atomic coordinates of the structure and mapped each protein chain to its UniProt identifier in the *E. coli* K-12 proteome. For each pair of ribosomal proteins, we computed the minimum Cα-Cα distance. We define direct interactions as protein pairs where this distance is below 8 Å, yielding a sparse ground-truth direct interaction matrix among the 46 ribosomal proteins.

Complex membership detection. We first ask whether ProteomeLM attention scores distinguish ribosomal proteins from the rest of the proteome. For each pair of ribosomal proteins (intra-complex) and for pairs consisting of one ribosomal and one non-ribosomal protein (inter-complex), we compute the mean attention score across all heads and layers. As shown in Figure S1A, intra-complex pairs receive significantly higher attention scores than inter-complex pairs. Quantitatively, ProteomeLM achieves an AUROC of 0.993 for distinguishing intra- from inter-complex pairs, confirming that the model reliably identifies co-complex membership at the proteome scale.

Direct interaction prediction. We next ask whether ProteomeLM attention coefficients can recover protein pairs in direct interaction *within* the ribosome. We evaluate the AUROC of each individual attention head for predicting the ground-truth direct interaction matrix among the 46 ribosomal proteins (Figure S1B). The best-performing head yields an AUROC of 0.671. To assess statistical significance, we perform a permutation test. Specifically, we use 1,000 permutations of the direct interaction labels, and consider the distribution of maximum AUROC across all heads in this case as the null distribution (Figure S1C). The observed maximum significantly exceeds the expected null maximum (0.615), with a p-value of 0.024, indicating that ProteomeLM encodes a statistically significant structural signal within this complex. However, signal is less strong than for complex membership detection (in line with Section 1). We further observe a weak but significant negative correlation between attention scores and physical distance among ribosomal protein pairs (Pearson correlation: -0.22), indicating that spatially close proteins tend to feature higher attention.

Interpretation. These results are consistent with the design and training objective of ProteomeLM. As a proteome-scale model trained to predict masked protein embeddings from genomic context, ProteomeLM learns functional co-dependencies between proteins, rather than direct interactions. The near-perfect complex membership detection (AUROC of 0.993) demonstrates that ProteomeLM robustly captures which proteins belong to the same complex, confirming the results of Section 1 on a specific example. The weaker

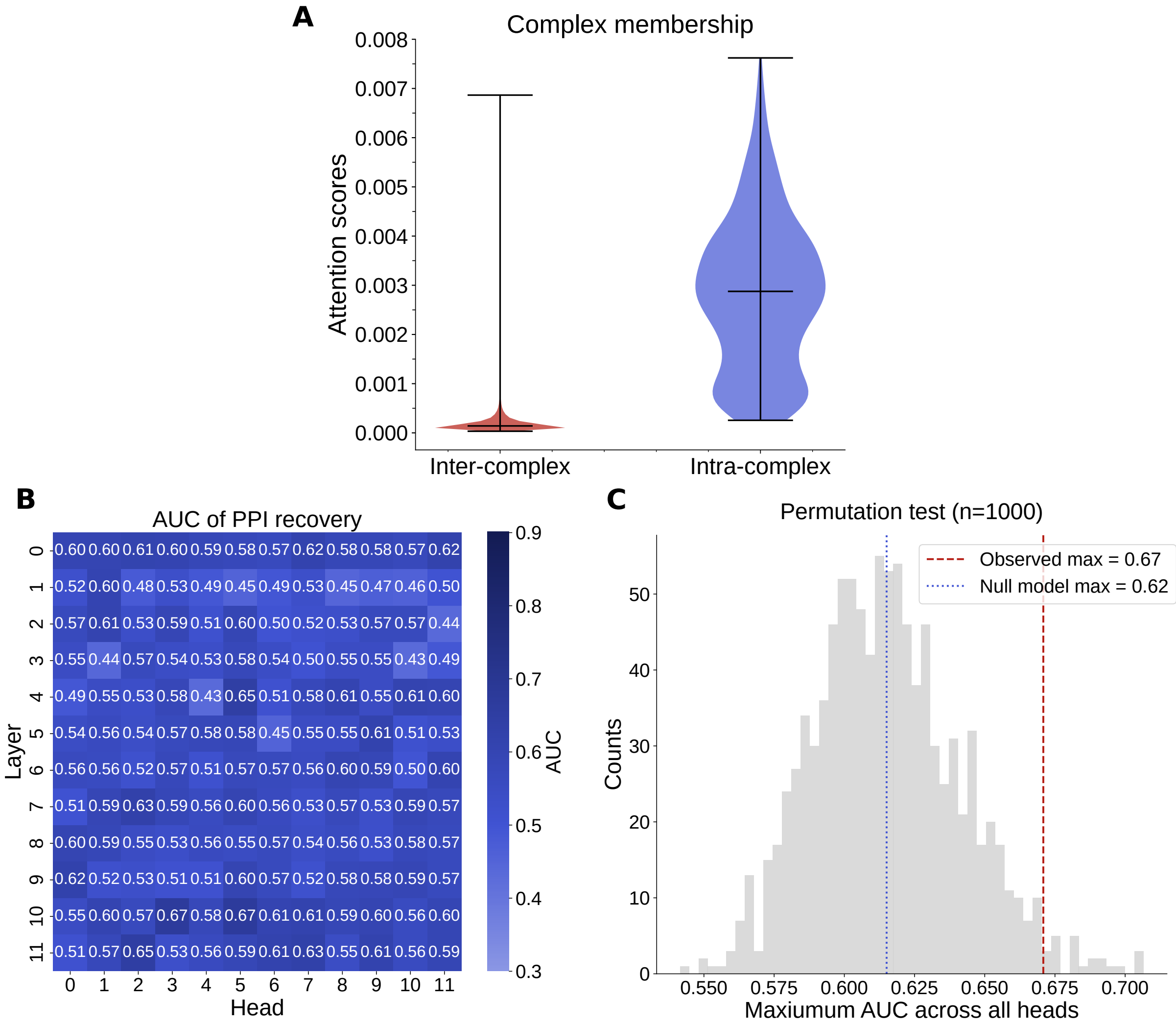

Figure S1: **Validation of ProteomeLM on the *E. coli* 70S ribosome (PDB: 7K00).** **(A)** Distribution (with extremes and median) of ProteomeLM attention scores for intra-complex (ribosomal–ribosomal) versus inter-complex (ribosomal–non-ribosomal) protein pairs. Intra-complex pairs receive substantially higher attention (membership AUROC: 0.993). **(B)** Per-head AUROC for predicting ground-truth direct interactions (minimum Cα-Cα distance < 8 Å) among the 46 mapped ribosomal proteins, for each attention head of ProteomeLM-M (12 layers, 12 heads). The best head achieves an AUROC of 0.671. **(C)** Permutation test ($n = 1{,}000$) for the maximum AUROC across all heads. The observed maximum (red dashed line, 0.671) significantly exceeds the expected null maximum (blue dotted line, 0.615), with a p-value of 0.024.

but significant intra-complex contact signal suggests that ProteomeLM encodes some information about the internal organization of complexes, and that there is room for improvement. Recall that ProteomeLM operates on whole-protein embeddings and does not have access to residue-level information. ProteomeLM can be employed as a fast and precise filter to identify candidate PPI pairs to be studied by more detailed but computationally much heavier models, such as Boltz [4, 5], or AlphaFold3 [6].

2.2 *S. cerevisiae* TRiC/CCT chaperonin

As another test case, we consider the eukaryotic TRiC/CCT chaperonin, which is a complex of 8 paralogous subunits (CCT1-8) arranged in a specific circular order [7]:

$$\text{CCT6} \to \text{CCT8} \to \text{CCT7} \to \text{CCT5} \to \text{CCT2} \to \text{CCT4} \to \text{CCT1} \to \text{CCT3} \to (\text{CCT6}).$$

This provides an interesting and challenging benchmark, because all 8 subunits are paralogs with high sequence similarity, all belong to the same complex, but only 8 out of 28 possible subunit pairs are in direct interaction within the ring. We processed the *S. cerevisiae* proteome through ProteomeLM and extracted attention coefficients for all possible pairs of CCT subunits.

Complex membership detection. We first ask whether ProteomeLM attention scores distinguish CCT complex members from non-members, as for the ribosome (Section 2.1). For each intra-complex pair (CCT-CCT, 28 pairs) and inter-complex pair (CCT-non-CCT, 8,000 pairs using 1,000 non-CCT proteins sampled uniformly at random), we compute the mean attention score across all heads and layers. As shown in Figure S2A, intra-complex pairs receive higher attention than inter-complex pairs, achieving a perfect membership classification. This confirms that ProteomeLM reliably identifies co-complex membership for this complex of paralogs, consistent with the results obtained in Sections 1 and 2.1.

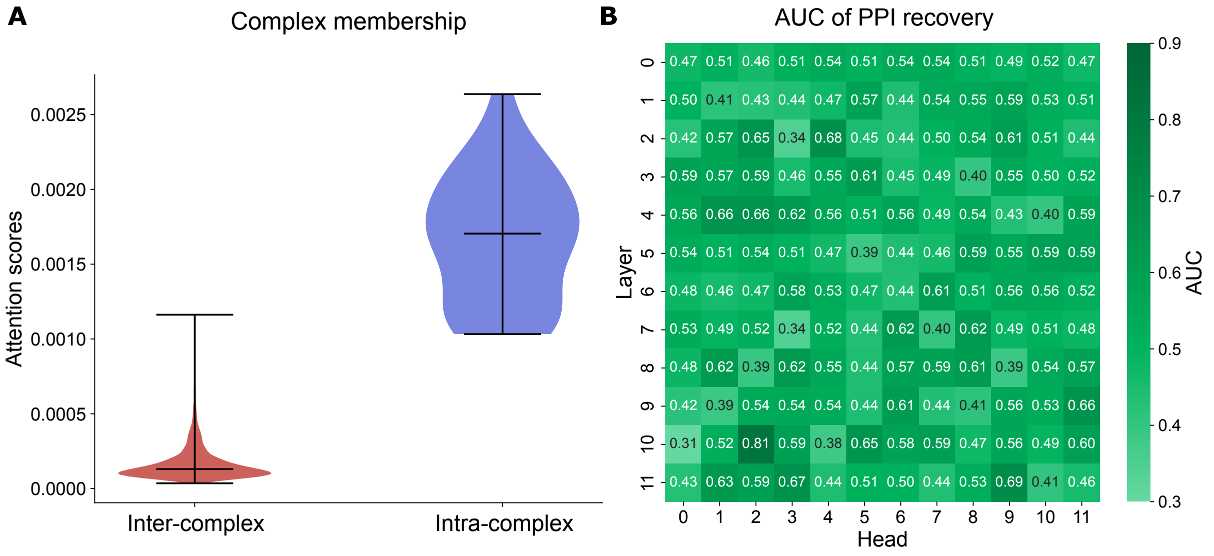

Figure S2: **ProteomeLM identifies TRiC/CCT complex membership but does not resolve the ring arrangement. (A)** Distribution of ProteomeLM attention scores for intra-complex (CCT–CCT) and inter-complex (CCT–non-CCT) protein pairs. Intra-complex pairs receive on average 8.4 times higher attention (Mann-Whitney U test p-value: 2.93×10^{-20}; membership AUROC = 1.00). **(B)** Per-head AUROC for discriminating adjacent from non-adjacent subunit pairs (8 adjacent out of 28 total), for each attention head of ProteomeLM-M (12 layers, 12 heads). The best head achieves an AUROC of 0.731, but a permutation test (n = 1,000) shows that this falls below the expected null maximum (0.793; p-value: 0.911), indicating no significant adjacent-pair signal.

Ring arrangement recovery. We next ask whether ProteomeLM can resolve the specific circular arrangement of subunits within the ring. Specifically, we evaluate whether individual attention heads discriminate adjacent from non-adjacent subunit pairs. The best-performing head achieves an AUROC of 0.731 (Figure S2B). However, a permutation test shows that this is not significant. We further enumerate all 5,040 circular permutations of the 8 subunits and score each by the total attention between consecutive pairs. The experimentally determined ring order [7] ranks 305th out of 5,040 (top 6.1%, $z = 1.58$), which does not reach statistical significance at conventional thresholds.

Interpretation. These results corroborate those obtained in Section 1: ProteomeLM reliably identifies which proteins belong to the same functional complex, but does not resolve fine-grained subunit arrangement within a complex as well. For TRiC/CCT, the 8 subunits descend from a common ancestor and occupy

structurally equivalent positions in the ring. Hence, from the perspective of evolutionary co-occurrence patterns, which ProteomeLM captures through proteome-level context, they are nearly interchangeable. The identification of direct PPI interactions is thus expected to be more challenging here than in the *E. coli* ribosome studied in Section 2.1, in line with our results.

3 Improvement of learned representations over input representations

ProteomeLM takes as input per-protein embeddings from ESM-C, which already encode evolutionary and functional information. An important question is whether ProteomeLM's attention coefficients simply encode similarities already present in the input embeddings, or whether they extract a more subtle signal. Furthermore, the functional encodings employed in ProteomeLM may encode further evolutionary information, since their construction involves a hierarchical averaging within orthology groups. This could potentially be relevant for the prediction of PPI, e.g. because interacting proteins have similar phylogenies [8, 9]. Here, we address these two points through comparative analyses.

3.1 Cosine similarity of input embeddings and functional encodings

For each species (*E. coli*, *S. cerevisiae*, *H. sapiens*), we compute the all-versus-all cosine similarity between ESM-C input embeddings and evaluate its ability to discriminate interacting from non-interacting pairs across three interaction types (direct, same complex, coexpression), using the benchmark described in Section 1. As shown in Table S3, ESM-C embeddings already capture information about PPI, achieving AUROC values of 0.61-0.75 for direct interactions and 0.74-0.83 for coexpression. In addition, we compute the all-versus-all cosine similarity between functional encodings. We obtain a weaker signal, with AUROC 0.58-0.70 for direct interaction and 0.46-0.68 for coexpression.

ProteomeLM's attention coefficients consistently and substantially outperform the baseline provided by cosine similarity between ESM-C embeddings. Specifically, the best individual ProteomeLM attention head achieves AUROC improvements of 0.09-0.16 over cosine similarity for direct interactions and 0.09-0.18 for coexpression (Table S3). Many attention heads outperform the ESM-C cosine similarity baseline (see Figure S3A), indicating that ProteomeLM learns nontrivial PPI-relevant information in its attention heads.

Table S3: **PPI prediction using cosine similarity of input embeddings, of functional encodings, and best ProteomeLM attention head.** Results are reported across three interaction types (see Section 1) in three species. All results are given in terms of AUROC.

**Species**	**Method**	**Direct**	**Complex**	**Coexpression**
E. coli	Input embedding	0.753	0.745	0.741
	Functional encoding	0.702	0.660	0.655
	Best ProteomeLM head	0.866	0.887	0.918
S. cerevisiae	Input embedding	0.688	0.671	0.782
	Functional encoding	0.642	0.612	0.680
	Best ProteomeLM head	0.782	0.826	0.942
H. sapiens	Input embedding	0.606	0.619	0.830
	Functional encoding	0.584	0.556	0.461
	Best ProteomeLM head	0.721	0.745	0.946

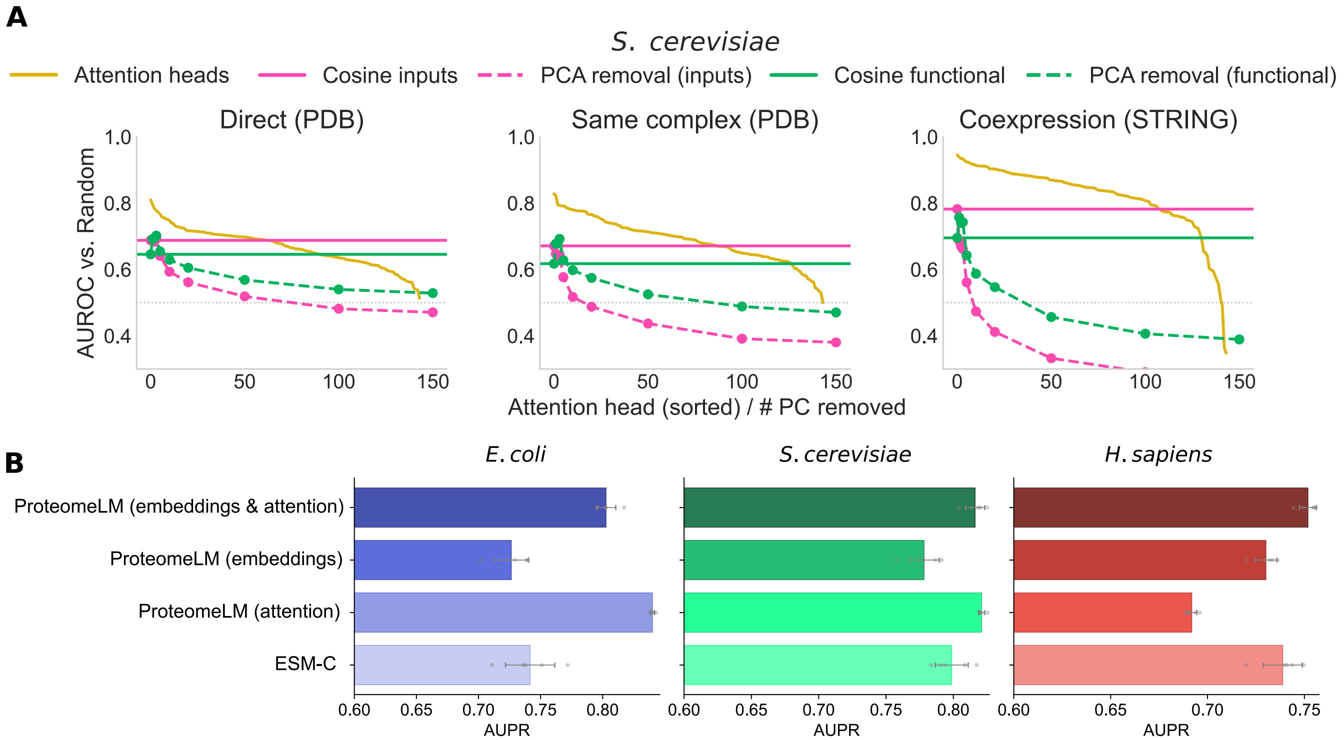

Figure S3: **ProteomeLM representations improve over input embeddings for PPI prediction.** **(A)** For each interaction type (direct, same complex, coexpression), we compare three unsupervised approaches: individual ProteomeLM-M attention heads sorted by AUROC, cosine similarity of ESM-C input embeddings, and cosine similarity of ProteomeLM functional encodings. For the latter two, we also perform progressive removal of the top k principal components. **(B)** AUPR of supervised multilayer perceptron classifiers trained on four feature sets, using ProteomeLM-M under sequence-similarity-controlled splits (40% identity): ESM-C embeddings alone, ProteomeLM attention alone, ProteomeLM embeddings alone, and the combination of ProteomeLM embeddings and attention. Error bars: standard deviation over 5 replicates (each shown with a gray marker).

3.2 PCA removal from the input similarity matrix

We perform a PCA decomposition of the all-versus-all cosine similarity matrix between ESM-C input embeddings, as well as of the one between functional encodings. In each of these two cases, we progressively project out the top k principal components (for $k = 0, 1, 5, 10, 20, \ldots, 148$), and we use the residual similarity scores to predict PPIs. As shown in Figure S3A, PPI prediction performance decreases rapidly with PC removal, falling below the original cosine baselines. This indicates that the dominant modes of pairwise similarity in the input space carry PPI-relevant signal. However, that signal is weaker than the one encoded in ProteomeLM attention heads. Thus, ProteomeLM's attention does not operate by simply filtering out top components of the input similarity matrix. We also note that the signal degrades more slowly in the functional encoding decomposition than in the input embedding decomposition.

3.3 Supervised evaluation with sequence-similarity-controlled splits

To further validate that ProteomeLM learns interaction-specific representations beyond sequence similarity, we constructed rigorous supervised benchmarks for three species using high-confidence interactions from PDB [10]. We applied sequence-similarity-aware train/validation/test splits: proteins were clustered at 40% sequence identity using MMseqs2 [11], and entire clusters were assigned to train/validation/test (50/20/30) via min-cut optimization [12] that minimizes lost cross-split interactions. This ensures that no protein

in the test set shares more than 40% sequence identity with any protein seen during training, thereby preventing homology-based information leakage.

We train simple multilayer perceptron classifiers (comprising one hidden layer with 64 units, and using ReLU activation functions) on this data, using different feature combinations:

- ESM-C embeddings alone (concatenated per-protein representations);
- ProteomeLM attention coefficients alone;
- ProteomeLM embeddings alone;
- ProteomeLM embeddings and attention coefficients.

As shown in Figure S3B, ProteomeLM attention coefficients alone outperform ESM-C embeddings in *E. coli* (AUPR of 0.80 vs. 0.74), and perform comparably in *S. cerevisiae.* Furthermore, the combination of ProteomeLM embeddings and attention coefficients consistently yields better performance than ESM-C embeddings across all three species. This result demonstrates the benefit of ProteomeLM features over the ESM-C baseline. It also confirms that ProteomeLM embeddings and attention coefficients carry complementary information. As elsewhere, we note that ProteomeLM performs particularly well on *E. coli*.

4 Comparison of compute requirements for human interactome scanning

Here, we focus on the specific problem of scanning the whole human interactome. A recent large-scale study applied DCA systematically to over 190 million *Homo sapiens* protein pairs [13]. We estimate the floating-point operations (FLOPs) required for this, as well as for each stage of our own screening with ProteomeLM. For this, we employ the peak FP32 throughput of relevant GPUs and the reported wall-clock runtimes. All values represent theoretical upper bounds. The results of the comparison below are summarized in Figure 3A.

DCA [13]. The Direct Coupling Analysis (DCA) step in Ref. [13] was performed over 1–2 months using between 50 and 100 GPUs, including NVIDIA RTX 6000, RTX 8000, A100, and A40. For our estimate, we assume:

- 75 concurrent GPUs over 45 days (i.e., 81,000 GPU-hours);
- Even distribution across the four GPU models;
- Peak FP32 throughput: 16.3 TFLOPS (RTX 6000 and RTX 8000), 19.5 TFLOPS (A100), and 37.4 TFLOPS (A40).

The resulting compute requirement is:

$$\text{Total FLOPs} \approx 6.5 \times 10^{21} \text{ FLOP (6.5 ZFLOP)}.$$

ProteomeLM training. Our model was trained on a single NVIDIA H100 SXM5 GPU during 72 hours. With a peak FP32 throughput of 67 TFLOPS, the total estimated compute is:

$$\text{Total FLOPs} = 67 \times 10^{12} \times 72 \times 3600 \approx 1.74 \times 10^{19} \text{ FLOP (17.4 EFLOP)}.$$

This value assumes uninterrupted training with full GPU utilization.

ProteomeLM inference. A 10-minute inference run was performed on an NVIDIA RTX A6000 GPU. With a peak FP32 throughput of 38.7 TFLOPS, the total compute estimated is:

$$\text{Total FLOPs} = 38.7 \times 10^{12} \times 600 \approx 2.32 \times 10^{16} \text{ FLOP (23 PFLOP)}.$$

5 Comparison of ProteomeLM-Ess with other gene essentiality prediction methods

In Figure S4, we provide a comparison of ProteomeLM-Ess with different state-of-the-art gene essentiality prediction methods. During the training of ProteomeLM-Ess, *E. coli* and *S. cerevisiae* were held out, and hence, none of the gene essentiality labels of these species were seen. Among the alternative methods considered, some of them used part of the labeled essentiality data from the two species of interest for training (see the list below for details). We observe in Figure S4 that ProteomeLM-Ess outperforms all other methods considered on *E. coli*. For *S. cerevisiae*, ProteomeLM-Ess outperforms all three methods that did not include labeled data from that species in training, by a substantial margin. However, the methods that included such data in training perform as well as or better than ProteomeLM-Ess. This suggests that their training set gives them an intrinsic advantage with respect to ProteomeLM-Ess. Overall, ProteomeLM-Ess is state of the art for *E. coli*, and also for *S. cerevisiae* among methods that do not include labeled data from that species in the training set. Note also that, among all methods considered here, Evo [14] and Evo 2 [15] are the only fully unsupervised methods, which explains their lower performance.

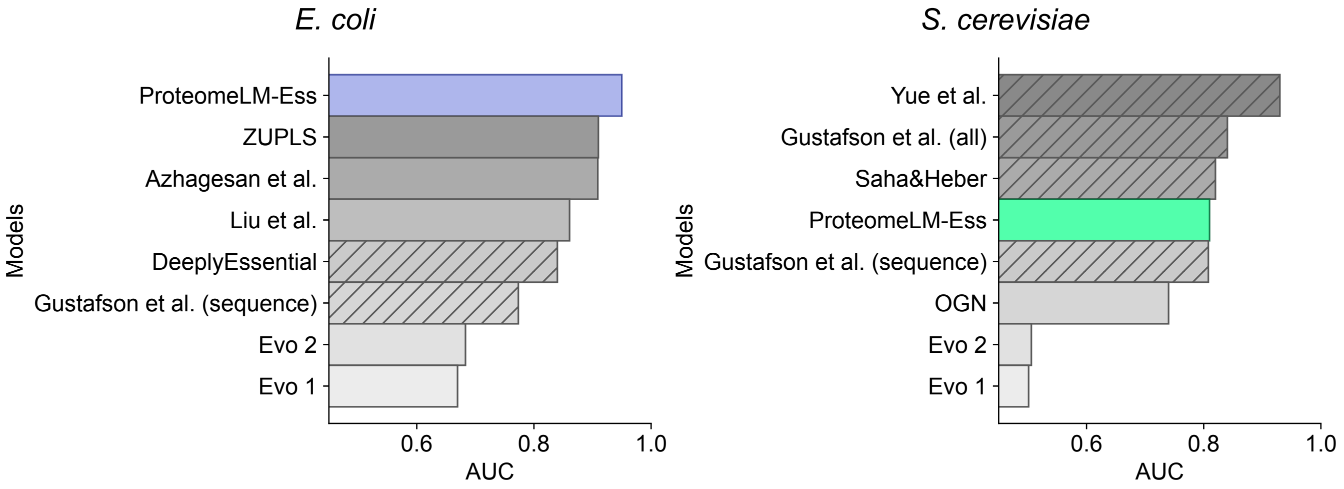

Figure S4: **Performance of ProteomeLM-Ess and of other methods for gene essentiality prediction.** The performance of ProteomeLM-Ess at predicting essential genes is compared to state-of-the-art methods for gene essentiality prediction [14–23], for *E. coli* (left) and *S. cerevisiae* (right). These other methods are briefly described in Section 5. Hatches indicate methods that use part of the organism's genes as labeled training data, contrary to ProteomeLM-Ess, where *E. coli* and *S. cerevisiae* were held out from the training set.

Figure S4 compares ProteomeLM-Ess to the following methods on *E. coli*:

- **ZUPLS**: Song et al. [16] use a partial least squares algorithm trained on sequence-derived features. The AU-ROC value shown in Figure S4 is the one they report when predicting *E. coli* essential genes by training the model on *B. subtilis* essential genes.
- **Azhagesan et al.**: Azhagesan et al. [17] train a random forest classifier on data from 27 prokaryotic organisms, using a combination of features derived from the sequence and from the protein-protein interaction network. The value of the AU-ROC shown in Figure S4 is the one they report for the leave-one-species-out validation for *E. coli*, i.e. when training the model on data from all organisms except *E. coli*.
- **Liu et al.**: Liu et al. [18] identify 40 sequence-derived features, and train a support vector machine classifier using data from 31 bacterial species. The AU-ROC value shown in Figure S4 is the one they report for the leave-one-species-out validation for *E. coli*.

- **DeeplyEssential**: Hasan and Lonardi [19] use data from 30 bacterial species and train a multilayer perceptron with 6 hidden layers using only sequence-derived features. The AU-ROC value shown in Figure S4 is an upper bound to the AU-ROC values they obtain for all Gram-negative bacteria. They aggregate data from all species and use 80% of genes as training set, 10% as validation set, and 10% as test set. Hence, part of the genes of *E. coli* are used for training.
- **Gustafson et al.**: Gustafson et al. [20] use sequence-derived and experimental features to classify essential genes in *E. coli* and *S. cerevisiae*. They separately train a naïve Bayes classifier for each species, using half of its essential genes and half of its non-essential genes as training set. For *E. coli*, they only use features derived from the sequence. The AU-ROC is computed on all genes, irrespective of whether they were used for training.
- **Evo 1**: Evo 1 [14] is a genomic foundation model that is trained in a self-supervised way on nucleotide sequences. Evo 1 has 7 billion parameters and a context length of up to 131,072 tokens. It was trained on prokaryotic sequences and was shown to be able to predict gene essentiality in a zero-shot fashion. Specifically, the model is given as input a 8192-nucleotide long context window centered in a protein-coding gene. Then a perturbation consisting of several stop codons (TAATAATAATAGTGA) is inserted at 12 bp after the beginning of the gene. The two sequences are passed through the model, which outputs a likelihood for each sequence. The ratio of the likelihoods is then used to predict essential genes. Nguyen et al. [14] used the model to make predictions for 56 bacterial species, including *E. coli*, and 2 phage species. The AU-ROC shown in Figure S4 was computed using the version of Evo 1 with 131k nucleotide context.
- **Evo 2**: Evo 2 [15] is a family of foundation models, with 7 billion or 40 billion parameters, trained at different context window lengths. It is trained using both prokaryotic and eukaryotic species. The gene essentiality prediction pipeline used for Evo 2 is identical to the one used for Evo 1. They report predictions only for prokaryotic species and for human lncRNAs (for which they use a different genetic perturbation, scrambling 100 bp portions of DNA at specific positions given by Cas13 guide sequence binding sites). The AU-ROC shown in Figure S4 was computed using the version of Evo 2 with 7B parameters and 8k nucleotide context.

Figure S4 compares ProteomeLM-Ess to the following methods on *S. cerevisiae*:

- **Yue et al.**: Yue et al. [21] use a deep learning method that combines different sources of information to predict gene essentiality. They separately construct embedding vectors for gene expression, PPI network, and subcellular localization. They then concatenate the three embedding vectors and use them as input for a one-layer neural network classifier with sigmoid activation. The model is used only on *S. cerevisiae* data, with 60% of the data used as training set, 20% as validation set and 20% for testing.
- **Gustafson et al.**: see above for method description. For *S. cerevisiae*, they both train a classifier using only sequence-derived features *(sequence)* and another one where these are combined with features obtained from experimental data *(all)*. Importantly, as for *E. coli*, some of the genes of *S. cerevisiae* are used for training and others for testing, and the AU-ROC shown in Figure S4 is computed on all genes, irrespective of whether they were used for training.
- **Saha&Heber**: Saha and Heber [22] use 1098 essential and 1098 non-essential genes of *S. cerevisiae* as training data for their classifier. They predict gene essentiality by combining a k nearest neighbor and a support vector machine classifier, which take as input features derived from the sequence, from protein-protein interaction data, as well as from the comparison between yeast data and data from other species.
- **OGN**: Zhang et al. [23] propose a mathematical model that combines measures of correlation in expression data with PPI network features. Their model outputs a parameter that is used to rank genes from most likely essential to least likely essential. They test their model on *S. cerevisiae* data.
- **Evo 1**: see above for method description. Recall that the model was trained and evaluated only on data from prokaryotic species. Hence, we performed our own evaluation of gene essentiality in *S.*

cerevisiae, using the same perturbation as for prokaryotes (see above). Note however that this is an out-of-distribution task for this model.

- **Evo 2**: see above for a description of the method. Recall that, contrary to Evo 1, the model's training set includes eukaryotes. However, gene essentiality prediction is not reported in Ref. [15] for these species. Hence, as for Evo 1, we performed our own evaluation of gene essentiality in *S. cerevisiae*. Note however that the choice of perturbation might not be optimal for eukaryotes.

6 Comparison of continuous and discrete functional encodings

Could the functional encoding used by ProteomeLM, which averages the ESM-C embeddings of proteins within each orthologous group, be replaced by a simpler discrete representation of orthologous group identity? To address this question, we trained an alternative version of ProteomeLM, which we refer to as ProteomeLM-Discrete, in which the continuous functional encoding is replaced by a learnable table indexed by OrthoDB group identifiers. Concretely, each orthologous group in the OrthoDB database [24] is assigned a unique integer index, and the model learns a fixed-dimensional embedding vector for each index from scratch during training. This design is analogous to the positional encoding usually employed in language models [25], where each position (here, each orthologous group identity) is represented by a learned vector. The rest of the architecture, including the transformer encoder, the masked language modeling objective, and the prediction heads, is kept identical to the standard ProteomeLM, in order to ensure a fair comparison.

Architecture details. We constructed an OrthoDB vocabulary comprising all orthologous group identifiers present in the training set, yielding a vocabulary of approximately 1.7 million entries. Each entry is mapped to a learnable embedding of dimension 1152 (matching the ESM-C embedding dimension), initialized with a normal distribution. An additional index is reserved for proteins with no known orthologous group assignment (`<UNK>`). For each protein, the orthologous group at the broadest taxonomic level is selected. ProteomeLM-Discrete was trained for 210 epochs under identical hardware and hyperparameter conditions as ProteomeLM-S (36M parameters), using the MSE loss, since the motivation for the polar loss (preventing collapse toward the functional encoding, see Methods and Section 7) does not apply when the functional encoding is a learned discrete vector unrelated to the target ESM-C embedding.

Results. We evaluate unsupervised PPI recovery by ProteomeLM-Discrete and compare it to the standard ProteomeLM-S on the benchmark distinguishing direct interactions, same-complex interactions and coexpression associations described in Section 1. For each model, we compute the AUROC of each individual attention head at distinguishing positive pairs from random pairs, across three species: *E. coli*, *S. cerevisiae*, and *H. sapiens*.

Figure S5 shows the per-head AUROC values, sorted in descending order, for both models across the three interaction types. ProteomeLM-Discrete (dashed lines) captures PPI signal across all species and interaction types, confirming that orthologous group identity alone provides a useful signal for learning inter-protein dependencies. However, our standard ProteomeLM with continuous functional encoding (solid lines) consistently outperforms ProteomeLM-Discrete. The gap is more pronounced in *E. coli*, where the best heads of ProteomeLM reach an AUROC of 0.87 for direct interactions, compared to 0.70 for ProteomeLM-Discrete. In *S. cerevisiae* and *H. sapiens*, ProteomeLM also possesses an advantage over ProteomeLM-Discrete, though the gap is smaller for coexpression associations than for direct or same-complex interactions.

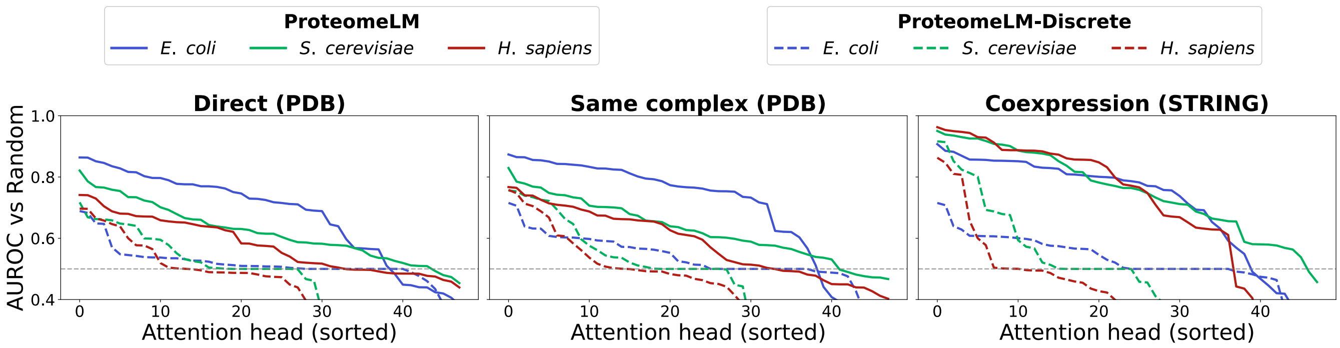

Figure S5: **Comparison of continuous and discrete functional encodings for unsupervised PPI prediction.** Per-head AUROC for distinguishing interacting from random protein pairs, sorted in descending order, for the standard ProteomeLM-S with continuous functional encoding and for ProteomeLM-Discrete with learnable OrthoDB group embeddings. Results are shown for three interaction types: direct interactions (PDB), same-complex interactions (PDB), and coexpression (STRING), see Section 1, across three species: *E. coli*, *S. cerevisiae*, and *H. sapiens*. Both models comprise 48 attention heads (6 layers × 8 heads).

Interpretation. These results indicate that our continuous functional encoding is beneficial beyond simply providing orthologous group identity. By encoding the average ESM-C embedding of each group, the functional encoding injects protein family-level sequence information into the model's input, which can be used by the model to better resolve inter-protein dependencies. The discrete encoding, while sufficient to capture broad co-occurrence patterns (as reflected in its reasonable coexpression recovery), lacks the fine-grained biochemical signal carried by ESM-C embeddings, which appears important in particular for distinguishing physical interactions. In addition, with a discrete encoding, the model cannot as efficiently transfer signal from one family to the other, which is critical when working with small families and with protein sequences with no assigned OrthoDB group. These results validate our design choice of using continuous, ESM-C-derived functional encodings.

7 Theoretical motivation of the polar loss used to train ProteomeLM

Let x denote the true protein embedding and $\bar{x}$ the functional encoding. We define the true residual vector as $r = x - \bar{x}$. Similarly, let $\hat{x}$ be the predicted embedding and $\hat{r} = \hat{x} - \bar{x}$ be the predicted residual.

Gradient collapse with the mean-squared-error loss. Let us write the standard mean-squared-error (MSE) loss between $\hat{x}$ and x as a function of the residuals:

$$\mathcal{L}_{\text{MSE}} \equiv \|\hat{x} - x\|^2 = \|\hat{r} - r\|^2 = \|\hat{r}\|^2 + \|r\|^2 - 2\|\hat{r}\|\|r\|\cos\theta, \tag{S1}$$

where $\|.\|$ denotes the Euclidean norm, and θ is the angle between $\hat{r}$ and r. The gradient of this loss with respect to the angular alignment θ between $\hat{r}$ and r is given by:

$$\frac{\partial \mathcal{L}_{\text{MSE}}}{\partial \theta} = 2\|\hat{r}\|\|r\|\sin\theta. \tag{S2}$$

Eq. S2 reveals that the signal for learning the correct direction is scaled by the product of the magnitudes of the true residual $\|r\|$ and of the predicted residual $\|\hat{r}\|$. In our setting, x and $\bar{x}$ are highly correlated, meaning that the true residual magnitude $\|r\|$ is small. Moreover, if the model defaults to the functional encoding for the protein embedding (i.e. $\hat{x} \to \bar{x}$), then $\|\hat{r}\| \to 0$. Consequently, the angular gradient in Eq. S2 vanishes. This prevents the model from learning the specific deviations of x from $\bar{x}$, and leads to the degenerate solution described in the main text.

Derivation of the polar loss via maximum likelihood estimation under magnitude-direction decoupling. In the maximum likelihood spirit, let us consider the true residual r as a random variable conditioned on the model prediction $\hat{r}$. To address the challenge identified above, let us further decouple the magnitude and the direction of the residual. To this end, we assume independent distributions for its radial (magnitude) and angular (directional) components in polar coordinates:

$$P(r|\hat{r}) \propto P_{\text{mag}}(\|r\| \mid \|\hat{r}\|)\, P_{\text{ang}}\left(\frac{r}{\|r\|} \,\middle|\, \frac{\hat{r}}{\|\hat{r}\|}\right). \tag{S3}$$

Let us now make simple assumptions on the explicit forms of the two distributions P_{mag} and P_{ang}.

Magnitude Component: We assume that the magnitude $\|r\|$ follows a normal distribution (denoted by $\mathcal{N}$) centered at the predicted magnitude $\|\hat{r}\|$ with variance σ^2:

$$P_{\text{mag}}(\|r\| \mid \|\hat{r}\|) \sim \mathcal{N}(\|\hat{r}\|, \sigma^2) \propto \exp\left[-\frac{(\|r\| - \|\hat{r}\|)^2}{2\sigma^2}\right] \implies -\log P_{\text{mag}}(\|r\| \mid \|\hat{r}\|) = C + \frac{(\|r\| - \|\hat{r}\|)^2}{2\sigma^2}\,, \tag{S4}$$

where C is a constant.

Directional Component: We further assume that the unit direction vector $u = r/\|r\|$ follows a von Mises-Fisher distribution (denoted by vMF) centered at the predicted direction $\hat{u} = \hat{r}/\|\hat{r}\|$ and with concentration parameter κ:

$$P_{\text{ang}}(u \mid \hat{u}) \sim \text{vMF}(\hat{u}, \kappa) \propto \exp(\kappa \hat{u} \cdot u) = \exp(\kappa \cos\theta) \implies -\log P_{\text{ang}}(u \mid \hat{u}) = C' - \kappa \cos\theta\,, \tag{S5}$$

where $\hat{u} \cdot u$ denotes the canonical scalar product of $\hat{u}$ and u, and C' is a constant.

Under the two assumptions above, the negative log-likelihood of $P(r|\hat{r})$ reads:

$$-\log P(r \mid \hat{r}) = C'' + \kappa(1 - \cos\theta) + \frac{(\|r\| - \|\hat{r}\|)^2}{2\sigma^2}, \tag{S6}$$

where C'' is a constant. Thus, under our assumptions, maximizing the likelihood of the data r under the model $\hat{r}$ amounts to minimizing a loss of the following form:

$$\mathcal{L}_{\text{Polar}} = \lambda_{\text{dir}}(1 - \cos\theta) + \lambda_{\text{mag}}(\|r\| - \|\hat{r}\|)^2, \tag{S7}$$

where λ_{dir} and λ_{mag} are hyperparameters corresponding to the inverse variances of the directional and magnitude noise, respectively. In practice, we use $\lambda_{\text{dir}} = \lambda_{\text{mag}} = 1$ in our polar loss, as a pragmatic choice. Crucially, the angular gradient of this loss is proportional to $\sin\theta$ but *independent* of $\|\hat{r}\|$. This decoupling prevents gradients for directional alignment from vanishing when the magnitude is small, thus avoiding the mode collapse identified above. Furthermore, our polar loss is minimized if and only if $\hat{x} = x$. Empirical validation of the polar loss against alternatives is shown in Figure 6D.

8 Supplementary figures

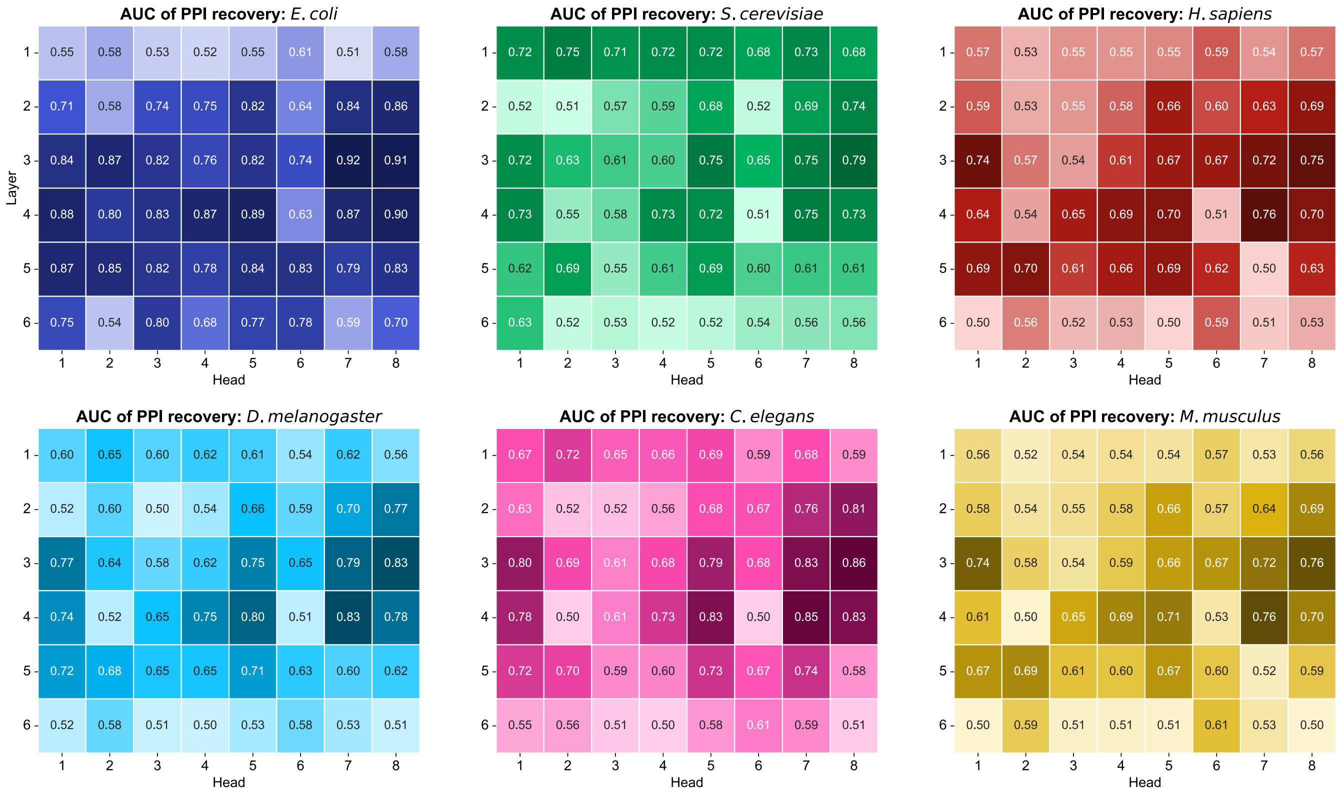

Figure S6: **Attention head-wise PPI recovery across species in ProteomeLM-S.** The area under the ROC curve (AUC) for protein-protein interaction (PPI) prediction using each individual attention head of ProteomeLM-S (6 layers, 8 heads) is reported across six species: *E. coli*, *S. cerevisiae*, *H. sapiens*, *D. melanogaster*, *C. elegans*, and *M. musculus*. Each heatmap shows the AUC for one species, with values computed separately for each attention head and layer. Attention heads from intermediate layers (particularly layer 3) consistently exhibit high predictive power, especially in *E. coli*. The first three panels (top) are also shown in Figure 2 and are reproduced here to facilitate inter-species comparison.

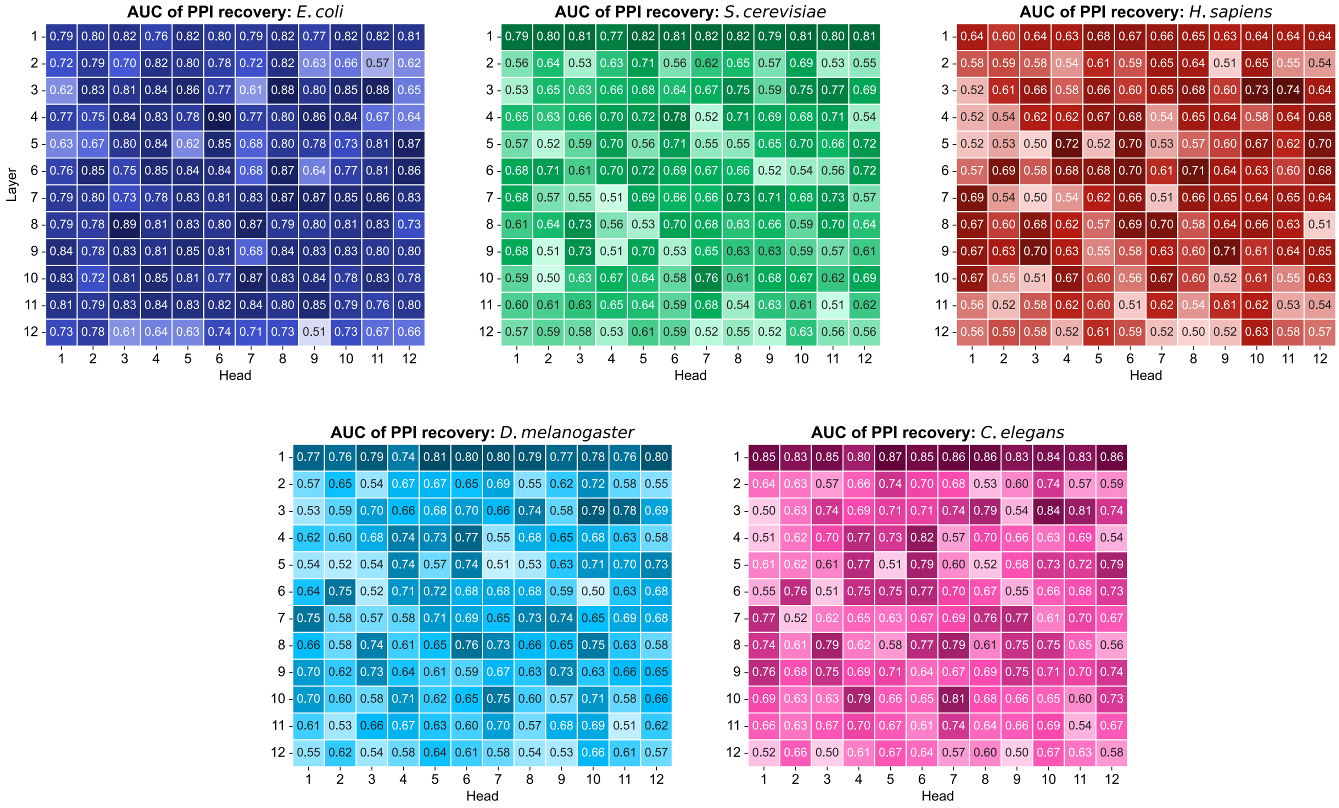

Figure S7: **Attention head-wise AUC of PPI recovery across species in ProteomeLM-M.** The AUC of protein-protein interaction (PPI) prediction is reported for each of the 12 attention head from all 12 layers of ProteomeLM-M (112M parameters), for five species: *E. coli*, *S. cerevisiae*, *H. sapiens*, *D. melanogaster*, and *C. elegans*. As in ProteomeLM-S (Figure S6), central layers tend to concentrate the most predictive heads. However, we note that the first layer is also a good predictor of PPI, in particular in eukaryotes. Note that the proteome of *M. musculus* is too long to allow the use of ProteomeLM-M.

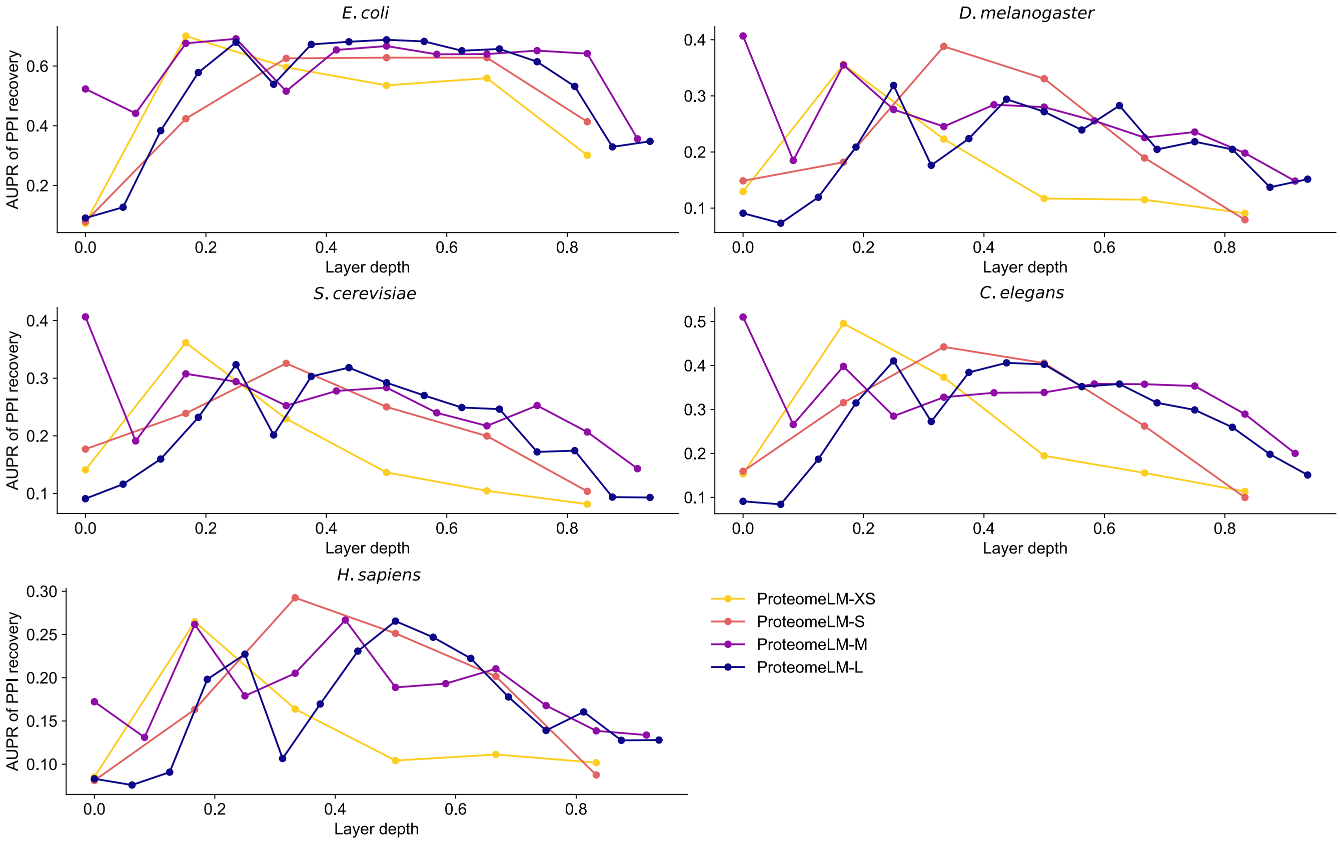

Figure S8: **Unsupervised PPI prediction by ProteomeLM across model sizes and layers.** The area under the precision-recall curve (AUPR) for unsupervised PPI prediction using summed attention scores is shown for each layer, across four model sizes (XS, S, M, L) and five species: *E. coli*, *S. cerevisiae*, *H. sapiens*, *D. melanogaster*, and *C. elegans*. To facilitate comparison between models with different sizes, the AUPR is plotted versus the normalized layer depth. Performance peaks at intermediate layers across species and model sizes.

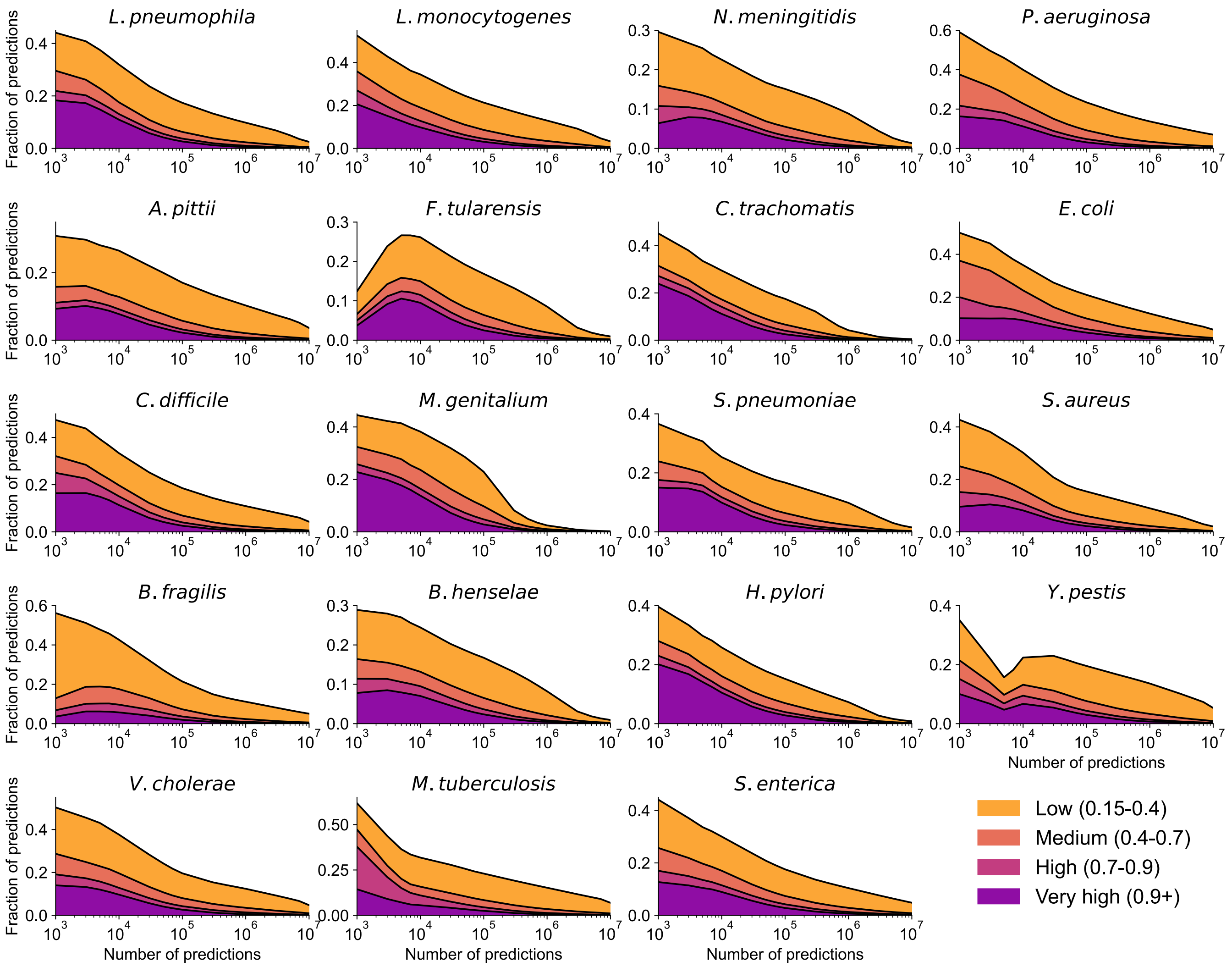

Figure S9: **Accuracy of fast PPI screening by ProteomeLM across 19 pathogens.** The fraction of top-scoring predicted PPI that correspond to known interactions in the STRING database is shown across 19 human bacterial pathogens. Predictions are sorted by the ProteomeLM classifier score, and their cumulative fraction in each of four bins of STRING confidence score is plotted as a function of the number of top-ranked predictions considered. Across most pathogens, a substantial fraction of high-scoring predictions correspond to known or high-confidence interactions. The 19 pathogens considered are the same as in Figure 3, and aggregated results are shown in Figure 3D.

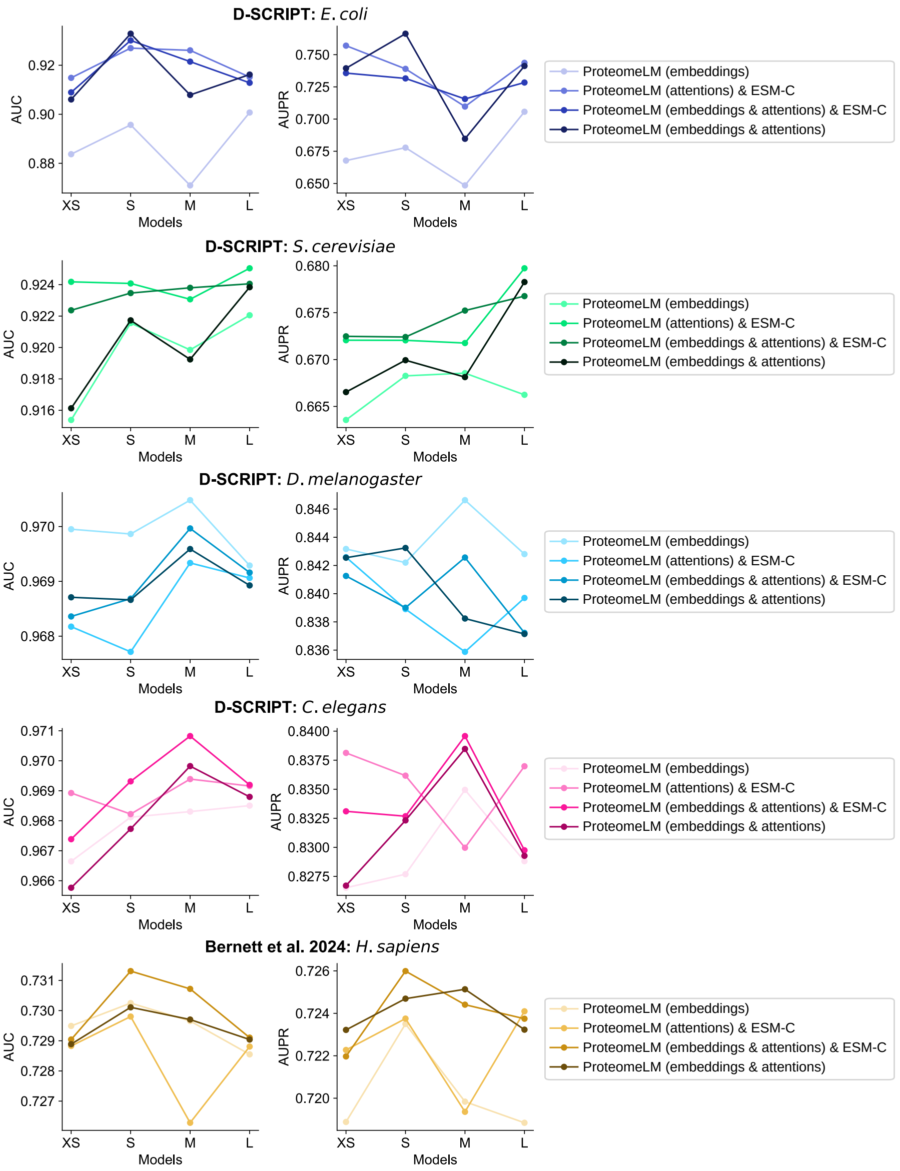

Figure S10: **Impact of embeddings and attention values on supervised PPI prediction.** Evaluation of supervised PPI prediction across four ProteomeLM model sizes (XS, S, M, L), on five benchmarks: the D-SCRIPT datasets for *E. coli*, *S. cerevisiae*, *D. melanogaster*, *C. elegans*, and the dataset from Ref. [26] for *H. sapiens*. For each of these benchmarks, both the AUC (left) and the AUPR (right) are reported using four different input feature configurations for PPI prediction: ProteomeLM embeddings only; ESM-C embeddings and ProteomeLM attention; ESM-C embeddings, ProteomeLM embeddings and ProteomeLM attention; and finally, ProteomeLM embeddings and ProteomeLM attention. The latter is the configuration we retained throughout, and called ProteomeLM-PPI. Indeed, we observe here that combining ProteomeLM embeddings and ProteomeLM attention yields the best or near-best performance across species and metrics.

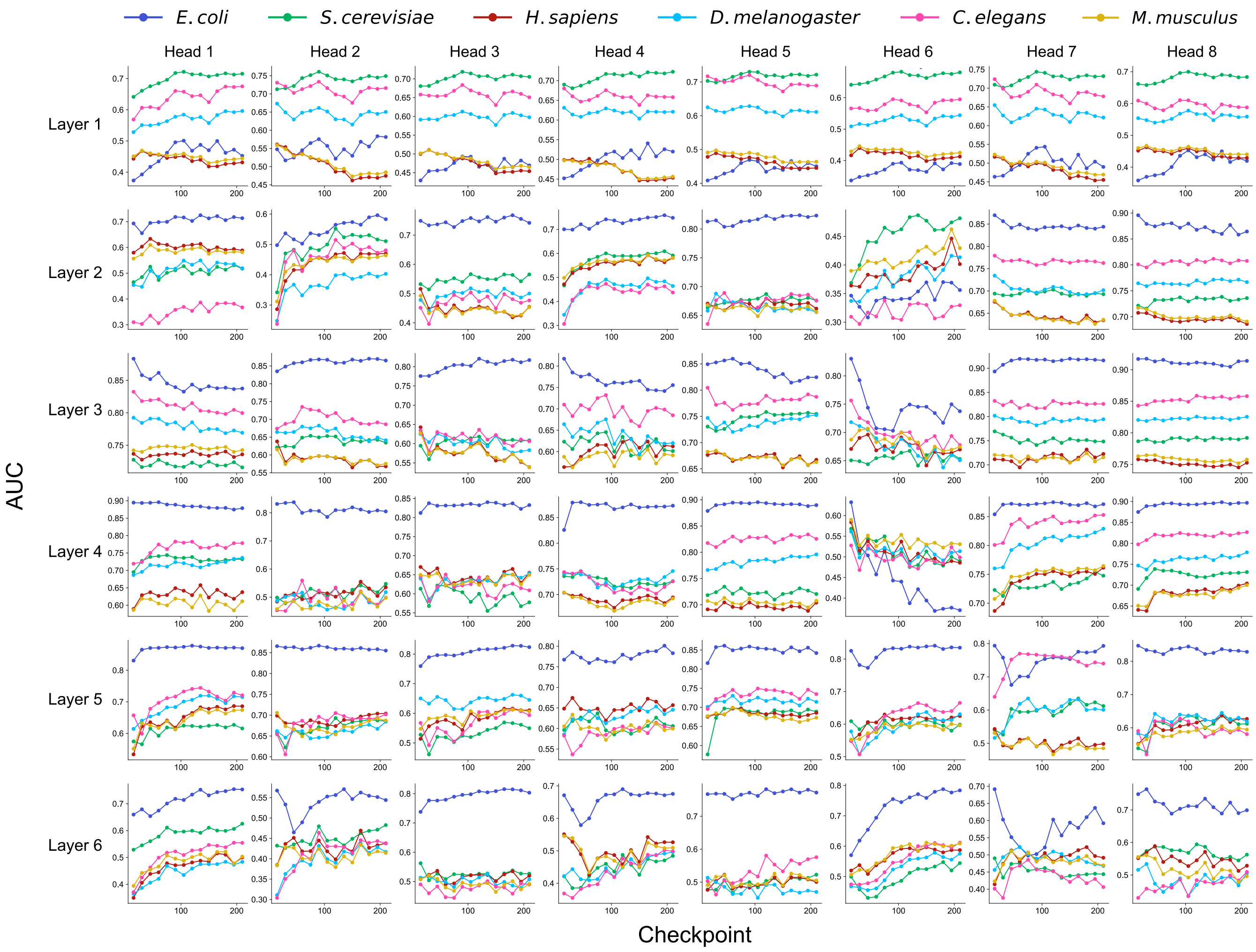

Figure S11: **Unsupervised recovery of PPI by ProtomeLM-S attention heads during training.** The area under the ROC curve (AUC) for unsupervised PPI recovery is shown versus training checkpoints for each attention head of ProteomeLM-S. Curves are shown separately for six species: *E. coli*, *S. cerevisiae*, *H. sapiens*, *D. melanogaster*, *C. elegans*, and *M. musculus*. Different heads exhibit different dynamics and specialization. Some become increasingly precise on *E. coli* (e.g. layer 6, head 3), while others (e.g. layer 1, head 1) are more specialized on eukaryotes. Many heads exhibit increasingly good PPI recovery along training. a

References

[1] M. Pellegrini, E. M. Marcotte, M. J. Thompson, D. Eisenberg, and T. O. Yeates. Assigning protein functions by comparative genome analysis: protein phylogenetic profiles. *Proc. Natl. Acad. Sci. USA*, 96(8):4285–4288, 1999.

[2] Damian Szklarczyk, Rebecca Kirsch, Mikaela Koutrouli, Katerina Nastou, Farrokh Mehryary, Radja Hachilif, Annika L Gable, Tao Fang, Nadezhda T Doncheva, Sampo Pyysalo, Peer Bork, Lars J Jensen, and Christian von Mering. The STRING database in 2023: protein–protein association networks and functional enrichment analyses for any sequenced genome of interest. *Nucleic Acids Res.*, 51(D1):D638–D646, 2022.

[3] Zoe L. Watson, Fred R. Ward, Raphaël Méheust, Omer Ad, Alanna Schepartz, Jillian F. Banfield, and Jamie Hd Cate. Structure of the bacterial ribosome at 2 Å resolution. *eLife*, 9:e60482, 2020.

[4] Jeremy Wohlwend, Gabriele Corso, Saro Passaro, Noah Getz, Mateo Reveiz, Ken Leidal, Wojtek Swiderski, Liam Atkinson, Tally Portnoi, Itamar Chinn, Jacob Silterra, Tommi Jaakkola, and Regina Barzilay. Boltz-1: Democratizing biomolecular interaction modeling. *bioRxiv*, page 2024.11.19.624167, 2024.

[5] Saro Passaro, Gabriele Corso, Jeremy Wohlwend, Mateo Reveiz, Stephan Thaler, Vignesh Ram Somnath, Noah Getz, Tally Portnoi, Julien Roy, Hannes Stark, David Kwabi-Addo, Dominique Beaini, Tommi Jaakkola, and Regina Barzilay. Boltz-2: Towards accurate and efficient binding affinity prediction. *bioRxiv*, page 2025.06.14.659707, 2025.

[6] Josh Abramson, Jonas Adler, Jack Dunger, Richard Evans, Tim Green, Alexander Pritzel, Olaf Ronneberger, Lindsay Willmore, Andrew J. Ballard, Joshua Bambrick, Sebastian W. Bodenstein, David A. Evans, Chia-Chun Hung, Michael O'Neill, David Reiman, Kathryn Tunyasuvunakool, Zachary Wu, Akvilė Žemgulytė, Eirini Arvaniti, Charles Beattie, Ottavia Bertolli, Alex Bridgland, Alexey Cherepanov, Miles Congreve, Alexander I. Cowen-Rivers, Andrew Cowie, Michael Figurnov, Fabian B. Fuchs, Hannah Gladman, Rishub Jain, Yousuf A. Khan, Caroline M. R. Low, Kuba Perlin, Anna Potapenko, Pascal Savy, Sukhdeep Singh, Adrian Stecula, Ashok Thillaisundaram, Catherine Tong, Sergei Yakneen, Ellen D. Zhong, Michal Zielinski, Augustin Žídek, Victor Bapst, Pushmeet Kohli, Max Jaderberg, Demis Hassabis, and John M. Jumper. Accurate structure prediction of biomolecular interactions with AlphaFold 3. *Nature*, 630(8016):493–500, 2024.

[7] Nir Kalisman, Christopher M. Adams, and Michael Levitt. Subunit order of eukaryotic TRiC/CCT chaperonin by cross-linking, mass spectrometry, and combinatorial homology modeling. *Proceedings of the National Academy of Sciences*, 109(8):2884–2889, 2012.

[8] F. Pazos and A. Valencia. Similarity of phylogenetic trees as indicator of protein–protein interaction. *Protein Eng. Des. Sel.*, 14(9):609–614, 2001.

[9] D. Ochoa, D. Juan, A. Valencia, and F. Pazos. Detection of significant protein coevolution. *Bioinformatics*, 31(13):2166–2173, 2015.

[10] H. M. Berman, J. Westbrook, Z. Feng, G. Gilliland, T. N. Bhat, H. Weissig, I. N. Shindyalov, and P. E. Bourne. The Protein Data Bank. *Nucleic Acids Res*, 28(1):235–242, 2000.

[11] Martin Steinegger and Johannes Söding. Clustering huge protein sequence sets in linear time. *Nature Communications*, 9(1):2542, 2018.

[12] Mechthild Stoer and Frank Wagner. A simple min-cut algorithm. *Journal of the ACM (JACM)*, 44(4):585–591, 1997.

[13] Jing Zhang, Ian R Humphreys, Jimin Pei, Jinuk Kim, Chulwon Choi, Rongqing Yuan, Jesse Durham, Siqi Liu, Hee-Jung Choi, Minkyung Baek, David Baker, and Qian Cong. Computing the human interactome. *bioRxiv*, page 2024.10.01.615885, 2024.

[14] Eric Nguyen, Michael Poli, Matthew G. Durrant, Brian Kang, Dhruva Katrekar, David B. Li, Liam J. Bartie, Armin W. Thomas, Samuel H. King, Garyk Brixi, Jeremy Sullivan, Madelena Y. Ng, Ashley Lewis, Aaron Lou, Stefano Ermon, Stephen A. Baccus, Tina Hernandez-Boussard, Christopher Ré, Patrick D. Hsu, and Brian L. Hie. Sequence modeling and design from molecular to genome scale with Evo. *Science*, 386(6723):eado9336, 2024.

[15] Garyk Brixi, Matthew G. Durrant, Jerome Ku, Michael Poli, Greg Brockman, Daniel Chang, Gabriel A. Gonzalez, Samuel H. King, David B. Li, Aditi T. Merchant, Mohsen Naghipourfar, Eric Nguyen, Chiara Ricci-Tam, David W. Romero, Gwanggyu Sun, Ali Taghibakshi, Anton Vorontsov, Brandon Yang, Myra Deng, Liv Gorton, Nam Nguyen, Nicholas K. Wang, Etowah Adams, Stephen A. Baccus, Steven Dillmann,

Stefano Ermon, Daniel Guo, Rajesh Ilango, Ken Janik, Amy X. Lu, Reshma Mehta, Mohammad R. K. Mofrad, Madelena Y. Ng, Jaspreet Pannu, Christopher Ré, Jonathan C. Schmok, John St John, Jeremy Sullivan, Kevin Zhu, Greg Zynda, Daniel Balsam, Patrick Collison, Anthony B. Costa, Tina Hernandez-Boussard, Eric Ho, Ming-Yu Liu, Thomas McGrath, Kimberly Powell, Dave P. Burke, Hani Goodarzi, Patrick D. Hsu, and Brian L. Hie. Genome modeling and design across all domains of life with Evo 2. *bioRxiv*, page 2025.02.18.638918, 2025.

[16] Kai Song, Tuopong Tong, and Fang Wu. Predicting essential genes in prokaryotic genomes using a linear method: ZUPLS. *Integrative Biology*, 6(4):460–469, 2014.

[17] Karthik Azhagesan, Balaraman Ravindran, and Karthik Raman. Network-based features enable prediction of essential genes across diverse organisms. *PLOS ONE*, 13(12):e0208722, 2018.

[18] Xiao Liu, Bao-Jin Wang, Luo Xu, Hong-Ling Tang, and Guo-Qing Xu. Selection of key sequence-based features for prediction of essential genes in 31 diverse bacterial species. *PLOS ONE*, 12(3):e0174638, 2017.

[19] Md Abid Hasan and Stefano Lonardi. DeeplyEssential: A deep neural network for predicting essential genes in microbes. *BMC Bioinformatics*, 21(14):367, 2020.

[20] Adam M. Gustafson, Evan S. Snitkin, Stephen C. J. Parker, Charles DeLisi, and Simon Kasif. Towards the identification of essential genes using targeted genome sequencing and comparative analysis. *BMC genomics*, 7:265, 2006.

[21] Yi Yue, Chen Ye, Pei-Yun Peng, Hui-Xin Zhai, Iftikhar Ahmad, Chuan Xia, Yun-Zhi Wu, and You-Hua Zhang. A deep learning framework for identifying essential proteins based on multiple biological information. *BMC Bioinformatics*, 23(1):318, 2022.

[22] Soma Saha and Steffen Heber. In silico prediction of yeast deletion phenotypes. *Genetics and molecular research*, 5(1):224–232, 2006.

[23] Xue Zhang, Wangxin Xiao, and Xihao Hu. Predicting essential proteins by integrating orthology, gene expressions, and PPI networks. *PLOS ONE*, 13(4):e0195410, 2018.

[24] Dmitry Kuznetsov, Fredrik Tegenfeldt, Mosè Manni, Mathieu Seppey, Matthew Berkeley, Evgenia V Kriventseva, and Evgeny M Zdobnov. OrthoDB v11: annotation of orthologs in the widest sampling of organismal diversity. *Nucleic Acids Res.*, 51(D1):D445–D451, 2022.

[25] Ashish Vaswani, Noam Shazeer, Niki Parmar, Jakob Uszkoreit, Llion Jones, Aidan N. Gomez, Łukasz Kaiser, and Illia Polosukhin. Attention is all you need. *Advances in Neural Information Processing Systems*, 30:5998–6008, 2017.

[26] Judith Bernett, David B Blumenthal, and Markus List. Cracking the black box of deep sequence-based protein–protein interaction prediction. *Briefings in Bioinformatics*, 25(2), 2024.
